# Conditional lethality and suppressor analysis of plasmid-based temperature-sensitive fabZ expression in *Pseudomonas aeruginosa*

**DOI:** 10.1016/j.jbc.2025.108553

**Published:** 2025-04-26

**Authors:** Zhenhua Chen, Junpeng Lu, Qinghai Tang, Zhili Yang

**Affiliations:** Systems Biology Laboratory, School of Marine Science and Technology, Zhejiang Ocean University, Zhoushan, Zhejiang, China

**Keywords:** plasmid-based ts-allele, essential gene, *fabZ*, suppressor, *Pseudomonas aeruginosa*

## Abstract

FabZ, a β-hydroxyacyl-acyl carrier protein dehydratase in the type II fatty acid synthesis pathway, is essential for the viability of *Pseudomonas aeruginosa* by ensuring proper fatty acid elongation and membrane stability. However, the precise genetic interactions between *fabZ* and lipid A biosynthesis genes, such as *lpxA* and *lpxC*, as well as the potential existence of other suppressor genes of *fabZ* in *P. aeruginosa*, remain unclear. To explore these genetic interactions and identify potential suppressor genes, we constructed a conditional *fabZ* mutant, *ΔfabZ*(p_ts-*fabZ*), by deleting the chromosomal *fabZ* gene and complementing it with a temperature-sensitive plasmid-borne copy. The *ΔfabZ*(p_ts-*fabZ*) mutant exhibited lethality and cell morphology defects at a restrictive temperature, confirming its essentiality. Genetic interaction analyses revealed that deletion of *lpxA* or *lpxC* failed to rescue *ΔfabZ*(p_ts-*fabZ*) lethality at restrictive temperature. Through suppressor screening, we isolated a mutant strain capable of rescuing *ΔfabZ* lethality and identified *lpxH* as the suppressor gene using genome resequencing. Further analysis revealed that the *fabZ* and *lpxH* double mutant (*ΔfabZΔlpxH*) produced odd-chain fatty acids, identified as pentadecanoic acid (C15:0) and heptadecanoic acid (C17:0) through fatty acid methyl ester analysis coupled with GC-MS, and supplementation with these fatty acids restored the growth and morphology of *ΔfabZ*(p_ts-*fabZ*) and *ΔlpxH*(p_ts-*lpxH*) mutants at restrictive temperature, suggesting their critical role in membrane stability. These results indicate that deletion of *lpxH* serves as a genetic suppressor of *ΔfabZ* lethality, highlighting a previously unrecognized compensatory mechanism involving odd-chain fatty acid synthesis essential for membrane stability in *P. aeruginosa*.

*Pseudomonas aeruginosa* is a Gram-negative bacterium widely found in natural environments, such as soil and water, but is also a major opportunistic pathogen responsible for severe infections in immunocompromised patients, particularly those with cystic fibrosis or burns (1). The frequent emergence of multidrug-resistant strains of *P. aeruginosa* has made it a pressing global health concern (2, 3). The World Health Organization has classified *P. aeruginosa* as a critical pathogen requiring new drug discovery efforts (4).

Essential genes in bacteria, whose inactivation leads to cell death, represent attractive targets for the development of novel antibacterial agents (5, 6, 7). Fatty acid synthesis, a vital metabolic pathway in bacteria, is an ideal target for antibiotic development due to its central role in maintaining cell membrane integrity and viability (8, 9, 10). Bacteria utilize the type II fatty acid synthesis (FASII) pathway, which consists of discrete, independently encoded enzymes. This makes FASII distinct from the multifunctional type I system found in humans, thereby minimizing potential off-target effects of FASII-targeting antibiotics (11, 12). Within this pathway, FabZ, a β-hydroxyacyl-acyl carrier protein (ACP) dehydratase, is critical for catalyzing the dehydration of β-hydroxyacyl-ACP intermediates, a key step in both saturated and unsaturated fatty acid biosynthesis (13). While FabA and FabZ share overlapping enzymatic functions, FabZ exhibits broader substrate specificity and plays a predominant role in saturated fatty acid elongation cycles (13, 14).

Many essential genes are suppressible (15, 16). In *Escherichia coli*, mutations in *fabZ* can suppress lipid A biosynthesis defects by rebalancing phospholipid and lipid A synthesis (17, 18). Lipid A serves as the hydrophobic anchor of lipopolysaccharide (LPS), a vital component of the outer membrane that maintains structural integrity and creates a permeability barrier in Gram-negative bacteria (19, 20, 21). Both *lpxA* and *lpxC*, essential genes in the lipid A biosynthesis pathway, are key targets of *fabZ*-mediated suppression (17, 18, 22). LpxA catalyzes the initial acylation step, while LpxC performs the first committed and rate-limiting deacetylation step in lipid A production (23, 24, 25). Perturbations in this pathway often lead to toxic intermediate accumulation, disrupting cellular homeostasis (18, 22). FabZ mitigates these imbalances by enhancing phospholipid synthesis, thereby stabilizing membranes and maintaining the balance between phospholipid and lipid A biosynthesis (18, 22). However, the genetic interactions between *fabZ* and lipid A biosynthesis genes, such as *lpxA* and *lpxC*, as well as other potential suppressor genes of *fabZ* in *P. aeruginosa*, have not been investigated and require further investigation due to their potential therapeutic significance.

To address these issues, we constructed a conditional *fabZ* mutant strain, *ΔfabZ*(p_ts-*fabZ*), by deleting the chromosomal *fabZ* gene and complementing it with a temperature-sensitive (ts) plasmid-expressing *fabZ* under its native promoter, as we have successfully employed in previous essential gene studies (26, 27, 28, 29). At the restrictive temperature of 42 °C, this strain failed to grow and exhibited oval-shaped cell morphology, confirming the essential role of *fabZ* in cell viability and membrane integrity. We further assessed the genetic interactions between *fabZ* and lipid A biosynthesis genes and identified a loss-of-function mutation in *lpxH* as a suppressor of *ΔfabZ* lethality. Together, our findings demonstrate that *fabZ* plays an essential role in regulating cell morphology and suggest potential links to the lipid A biosynthesis gene *lpxH* in maintaining cellular homeostasis.

## Results

### Construction of *ΔfabZ*(p_ts-*fabZ*) and analysis of its ts lethality in *P. aeruginosa*

**Figure 1:**
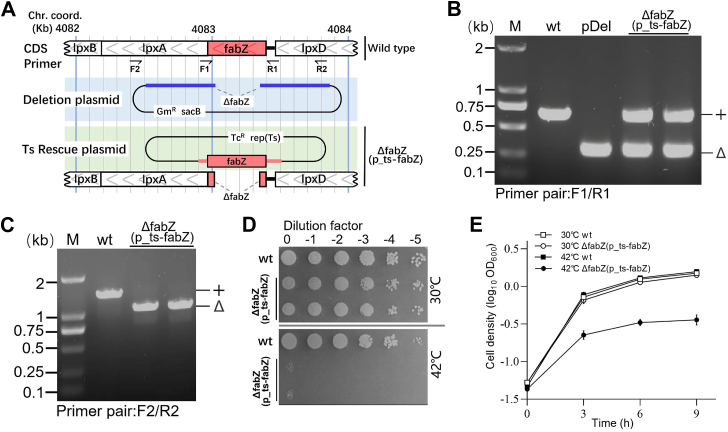
**Growth defect of *ΔfabZ*(p_ts-*fabZ*) at restrictive temperature.***A,* schematic of the *fabZ* deletion cassette (*blue*) in the deletion plasmid and the complementary *fabZ* allele (*red*) in the ts rescue plasmid. *ΔfabZ*(p_ts-*fabZ*) contains a chromosomal *fabZ* deletion (*ΔfabZ*) and a native promoter-controlled complementary allele on the ts plasmid. Primer locations (F1, R1, F2, and R2) for detecting WT and deletion alleles are indicated on the schematic. *B* and *C,* PCR analysis of *fabZ* alleles using primer pairs F1/R1 and F2/R2 to detect the chromosomal *ΔfabZ* allele in *ΔfabZ*(p_ts-*fabZ*) strains. *D,* spot-plating assay showing growth of WT and ts mutant strains at 30 °C and 42 °C. *E,* growth curves of *ΔfabZ*(p_ts-*fabZ*) at 30 °C and 42 °C, with time (h) on the *x*-axis and cell density (log_10_ A_600_) on the *y*-axis. ts, temperature-sensitive.

To examine the genetic function of the essential gene *fabZ* in *P. aeruginosa*, we constructed a plasmid-based ts mutant, *ΔfabZ*(p_ts-*fabZ*), using the three-step protocol previously developed (26, 27, 28, 29). Briefly, the construction of deletion plasmid carrying the *fabZ* deletion allele (*ΔfabZ*), along with the ts rescue plasmid p_ts-*fabZ* containing the WT *fabZ* allele under native promoter control, is illustrated in Figure 1*A*. PCR analysis confirmed the absence of the WT *fabZ* allele in the deletion plasmid (Fig. 1*B*, lane pDel), while both the *fabZ* deletion allele (*ΔfabZ*) and the WT allele (*fabZ*+) were detected in the *ΔfabZ*(p_ts-*fabZ*) mutant strain (Fig. 1*B*, lanes ts) using primer pair F1/R1(Fig. 1*A*, line Primer). Additionally, PCR analysis using primer pair F2/R2 confirmed that the chromosomal *fabZ* gene was successfully deleted in the *ΔfabZ*(p_ts-*fabZ*) strain, with no WT *fabZ* detected (Fig. 1*C*, lanes ts). To assess the mutant's growth phenotype, spot-plating assays were performed with serial dilutions of cells. The *ΔfabZ*(p_ts-*fabZ*) mutant was unable to grow at the restrictive temperature of 42 °C, confirming the essential role of *fabZ* for *P. aeruginosa* growth (Fig. 1*D*). The growth curve analysis corroborated the findings from the spot-plating assay (Fig. 1*E*), further validating the essentiality of *fabZ* in supporting *P. aeruginosa* growth.

### *fabZ* is essential for maintaining rod-shaped morphology in *P. aeruginosa*

**Figure 2:**
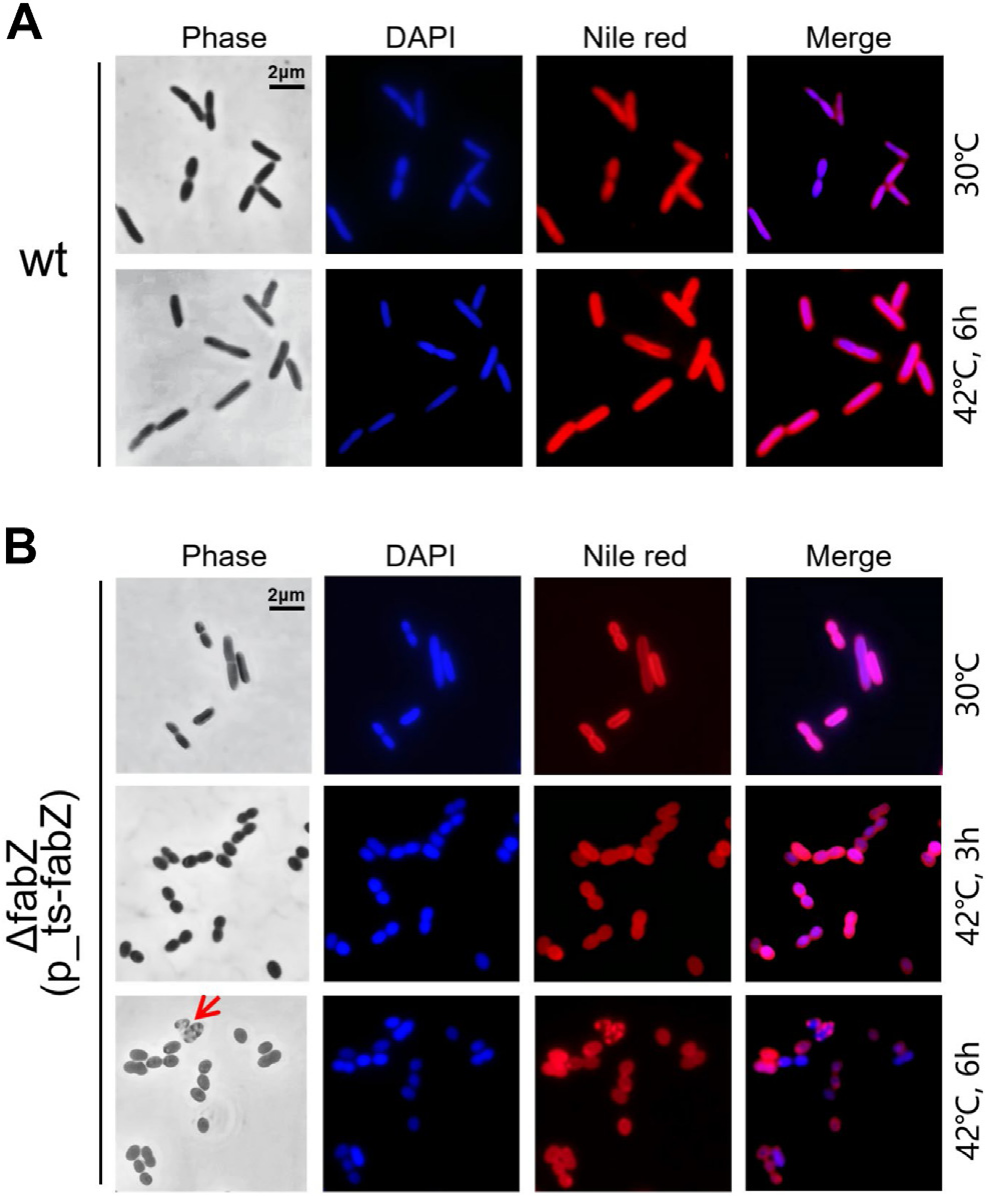
**Morphological changes in *ΔfabZ*(p_ts-*fabZ*) cells at 42 °C observed under fluorescence microscopy.** Cells were stained with DAPI to visualize DNA (*blue*) and Nile *red* to highlight the plasma membrane (*red*). The scale bar represents 2 μm. *A,* morphology of WT cells grown at 30 °C and 42 °C. *B,* morphology of *ΔfabZ*(p_ts-*fabZ*) cells grown at 30 °C and 42 °C. DAPI, 4′,6-diamidino-2-phenylindole; ts, temperature-sensitive.

The *fabZ* gene encodes an enzyme crucial for bacterial fatty acid synthesis, which is vital for maintaining cell membrane integrity and proper cell shape. To investigate the role of *fabZ* in maintaining cell morphology in *P. aeruginosa*, microscopic analysis was conducted to examine the morphological characteristics of *ΔfabZ*(p_ts-*fabZ*) cells under permissive (30 °C) and restrictive temperature. The results demonstrated that WT cells consistently maintained a rod-shaped morphology, with no observable differences between cells grown at 30 °C and 42 °C (Fig. 2*A*). Similarly, *ΔfabZ*(p_ts-*fabZ*) cells exhibited a rod-shaped morphology identical to the WT at 30 °C (Fig. 2*B*). However, upon shifting to the restrictive temperature of 42 °C for 3 h, where *fabZ* function is progressively lost, the *ΔfabZ*(p_ts-*fabZ*) cells began to adopt an oval shape (Fig. 2*B*), which could result from impaired cell membrane synthesis. By 6 h, in addition to oval-shaped cells, ghost cells or lysed cells were also observed (Fig. 2*B*, bottom row, indicated by arrows). These findings suggest that *fabZ* is crucial for maintaining the rod-shaped morphology of *P. aeruginosa*.

### *fabA* overexpression cannot compensate for the essential role of *fabZ* in *P. aeruginosa*

**Figure 3:**
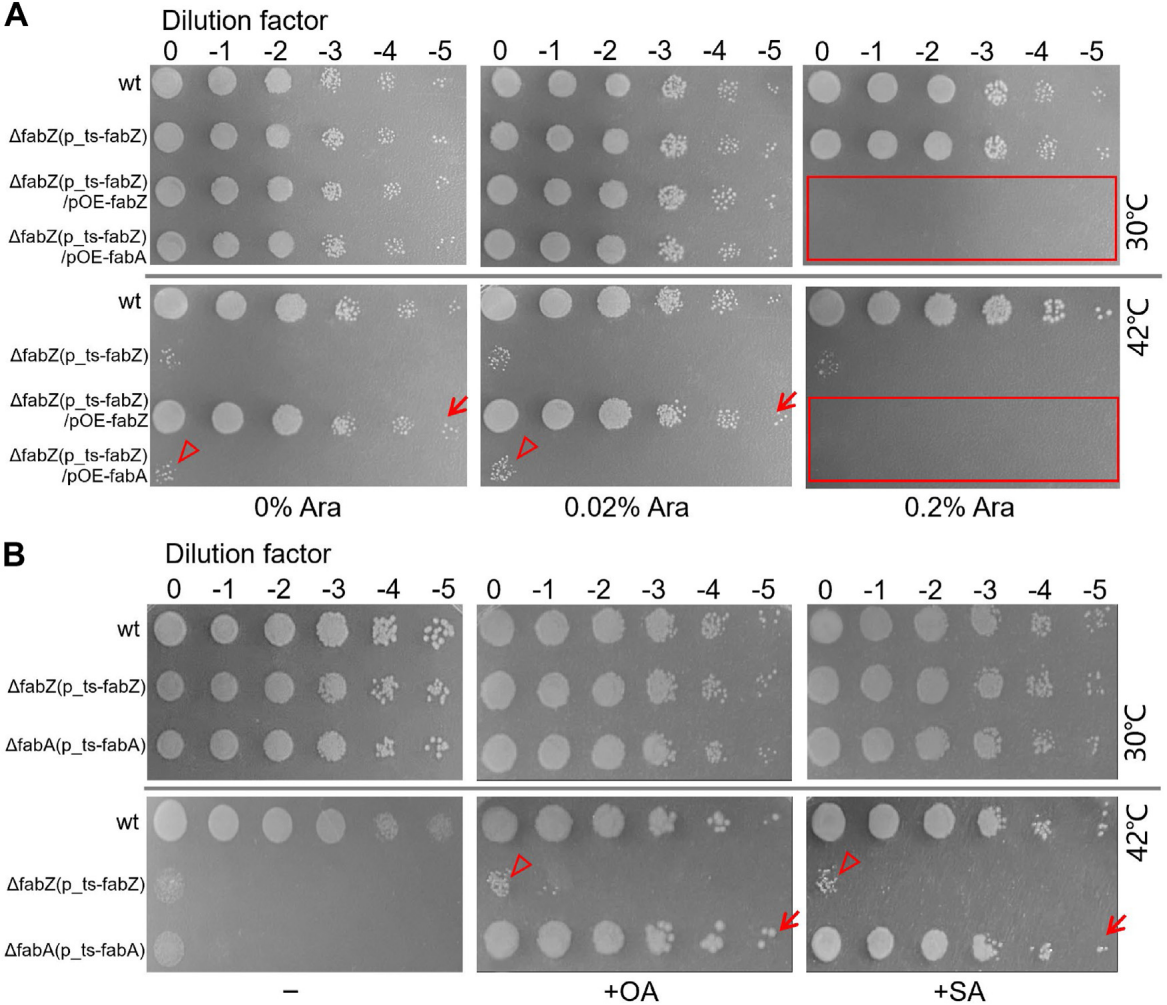
**Functional complementation analysis of *fabA* and *fabZ*.***A,* spot-plating assay of wt, *ΔfabZ*(p_ts-*fabZ*), *ΔfabZ*(p_ts-*fabZ*)/pOE-*fabZ*, and *ΔfabZ*(p_ts-*fabZ*)/pOE-*fabA* strains on LB agar supplemented with different concentrations of arabinose (0%, 0.02%, and 0.2%). Plates were incubated at 30 °C and 42 °C, respectively. *B,* spot-plating assay of wt, *ΔfabA*(p_ts-*fabA*), and *ΔfabZ*(p_ts-*fabZ*) mutants on LB agar with or without supplementation of oleic acid (OA) or stearic acid (SA) at 30 °C and 42 °C. ts, temperature-sensitive.

FabA and FabZ are both β-hydroxyacyl-ACP dehydratases that play essential roles in bacterial fatty acid biosynthesis (13). While FabA primarily targets medium-chain substrates, FabZ is more efficient with shorter chain substrates (13). To investigate whether *fabA* overexpression can compensate for the growth defect of *ΔfabZ*(p_ts-*fabZ*) mutant in *P. aeruginosa* at 42 °C, we constructed the pOE-*fabA* plasmid with *fabA* expression driven by the arabinose-inducible P_BAD_ promoter (30) in the multihost pBBR1MCS-5 plasmid (31), resulting in pOE-*fabA* constructs. As a control, the pOE-*fabZ* plasmid was similarly constructed. Spot-plating assay indicated that *fabZ* gene overexpression at leakage expression levels (no arabinose) or mild induction levels (0.02% arabinose) could restore the growth of *ΔfabZ*(p_ts-*fabZ*)/pOE-*fabZ* at 42 °C (Fig. 3*A*, see arrows). However, similar induction levels of *fabA* overexpression from the pOE-*fabA* plasmid did not restore the growth of *ΔfabZ*(p_ts-*fabZ*)/pOE-*fabA* at 42 °C (Fig. 3*A*, see arrowheads), indicating that *fabA* cannot functionally replace *fabZ* in *P. aeruginosa*. Additionally, strong induction (0.2% arabinose) of both *fabA* and *fabZ* overexpression impeded cell growth (Fig. 3*A*, see rectangles). This might be due to excessive FabA or FabZ activity depleting their substrate, 3-hydroxyacyl-ACP, which limits the synthesis of lipid A—a critical LPS component of the Gram-negative bacterial outer membrane (32).

To further validate the functional relationship between *fabA* and *fabZ*, we supplemented the growth medium with oleic acid (OA) or stearic acid (SA) (27). Consistent with our previous findings, oleic acid and SA supplementation restored the growth of *ΔfabA*(p_ts-*fabA*) mutants at 42 °C (27) (Fig. 3*B*, see arrows). However, this supplementation failed to rescue *ΔfabZ*(p_ts-*fabZ*) mutants at 42 °C (Fig. 3*B*, see arrowheads), underscoring the indispensable role of *fabZ* in maintaining fatty acid biosynthesis and cell growth. These results suggest that while *fabA* and *fabZ* both participate in fatty acid biosynthesis, their functions are distinct and noninterchangeable in *P. aeruginosa*.

### Deletion of *lpxA* or *lpxC* fails to suppress *ΔfabZ*(p_ts-*fabZ*) lethality in *P. aeruginosa*

LPS is a major component of the outer membrane in Gram-negative bacteria, comprising three parts: lipid A, core oligosaccharide, and O-antigen polysaccharide. Lipid A serves as the anchor of LPS and is essential for bacterial viability (19, 20, 21), since its absence disrupts LPS synthesis, leading to cell death. In *E. coli* and *Klebsiella pneumoniae*, it has been reported that disruption of *fabZ* suppresses the growth defect of *lpxA* or *lpxC* mutants that are unable to synthesize lipid A (17, 18, 22, 33).

**Figure 4:**
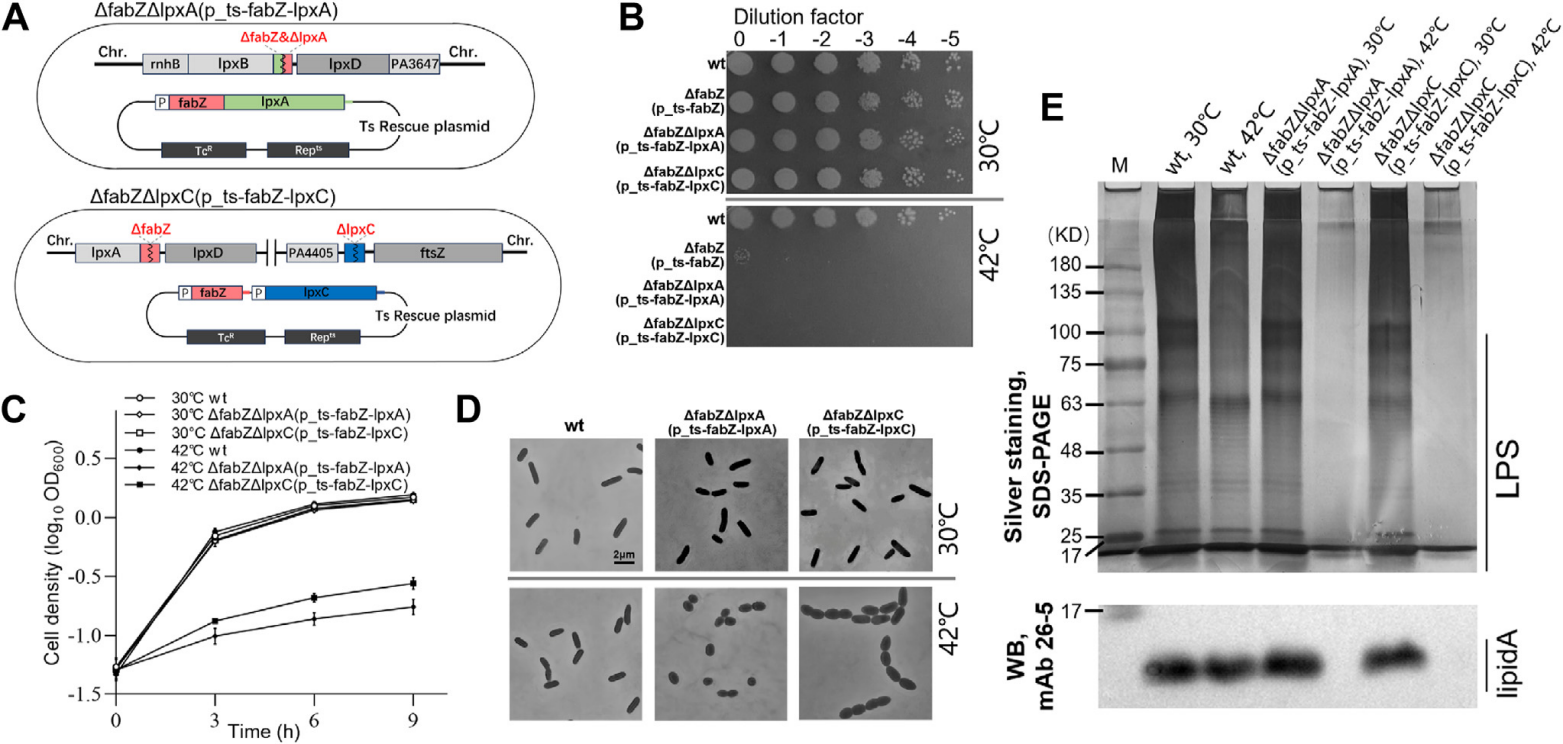
**Construction and phenotypic analysis of *ΔfabZΔlpxA*(p_ts-*fabZ*-*lpxA*) and *ΔfabZ*Δ*lpxC*(p_ts-*fabZ*-*lpxC*) at a restrictive temperature.***A,* schematic map of *ΔfabZΔlpxA*(p_ts-*fabZ*-*lpxA*) and *ΔfabZΔlpxC*(p_ts-*fabZ*-*lpxC*). *ΔfabZΔlpxA*(p_ts-*fabZ*-*lpxA*) contains two deletion allele, *ΔfabZ* and *ΔlpxA,* which are contiguous and belong to the same operon, on the chromosome and a native promoter-controlled complementary copy, *fabZ* and *lpxA*, on the ts rescue plasmid. *ΔfabZΔlpxC*(p_ts-*fabZ*-*lpxC*) is analogous, with *ΔfabZ* and *ΔlpxC* on the chromosome and *fabZ* and *lpxC* under native promoter control on the ts rescue plasmid. *B,* spot-plating assay showing growth of WT and ts mutant strains at 30 °C and 42 °C. *C,* growth curve analysis of the WT and ts mutant strains at 30 °C and 42 °C. *D,* microscopic analysis of the WT and ts mutant strains at 30 °C and 42 °C. *E,* silver-stained SDS-PAGE gel and Western blot image. LPS components including lipid A, core oligosaccharide, and O-antigen are separated on an 8% SDS-PAGE gel (*up panel*). Molecules derived from the *bottom* 8% SDS-PAGE gel are probed with mAb 26-5 against lipid A (*down panel*). ts, temperature-sensitive; LPS, lipopolysaccharide.

To determine whether similar suppressions occur in *P. aeruginosa*, we constructed plasmid-based dual-gene ts mutants, *ΔfabZΔlpxA*(p_ts-*fabZ*-*lpxA*) and *ΔfabZΔlpxC*(p_ts-*fabZ*-*lpxC*), using our three-step protocol (Fig. 4*A*, see Experimental procedures). PCR analysis confirmed that *fabZ* and *lpxA* were deleted from the chromosome (labeled as “Δ”) and the WT alleles were present on the ts rescue plasmid (labeled as “+”) in the *ΔfabZΔlpxA*(p_ts-*fabZ*-*lpxA*) strain (Fig. S1, *A* and *B*). Similarly, the *ΔfabZΔlpxC*(p_ts-*fabZ-lpxC*) strain showed the deletion of *fabZ* and *lpxC* on the chromosome while harboring the WT alleles on the ts rescue plasmid (Fig. S1, *C* and *D*).

Spot-plating assays revealed that neither *ΔfabZΔlpxA*(p_ts-*fabZ*-*lpxA*) nor *ΔfabZΔlpxC*(p_ts-*fabZ-lpxC*) strains were able to grow at the restrictive temperature of 42 °C, indicating that the lethality of *ΔfabZ* cannot be suppressed by *lpxA* or *lpxC* deletion in *P. aeruginosa* (Fig. 4*B*). Growth curve analyses corroborated these findings, showing that *ΔfabZΔlpxA*(p_ts-*fabZ*-*lpxA*) and *ΔfabZΔlpxC*(p_ts-*fabZ-lpxC*) strains exhibited severely impaired growth at 42 °C (Fig. 4*C*). Microscopic analysis revealed that *ΔfabZΔlpxA*(p_ts-*fabZ*-*lpxA*) and *ΔfabZΔlpxC*(p_ts-*fabZ-lpxC*) mutants exhibited oval-shaped cell morphology at 42 °C, indicating disrupted membrane functionality (Fig. 4*D*).

Furthermore, Western blot analysis demonstrated the absence of lipid A in the *ΔfabZΔlpxA*(p_ts-*fabZ-lpxA*) and *ΔfabZΔlpxC*(p_ts-*fabZ-lpxC*) mutants at 42 °C (Fig. 4*E*, bottom panel), which was attributed to the deletion of *lpxA* or *lpxC*, both essential for lipid A synthesis. Silver staining also indicated that in the absence of lipid A, which serves as a membrane anchor, other LPS components were not detected in the mutants (Fig. 4*E*, top panel).

### A suppressor restores cell growth but not cell morphology of *ΔfabZ*(p_ts-*fabZ*) at a restrictive temperature

**Figure 5:**
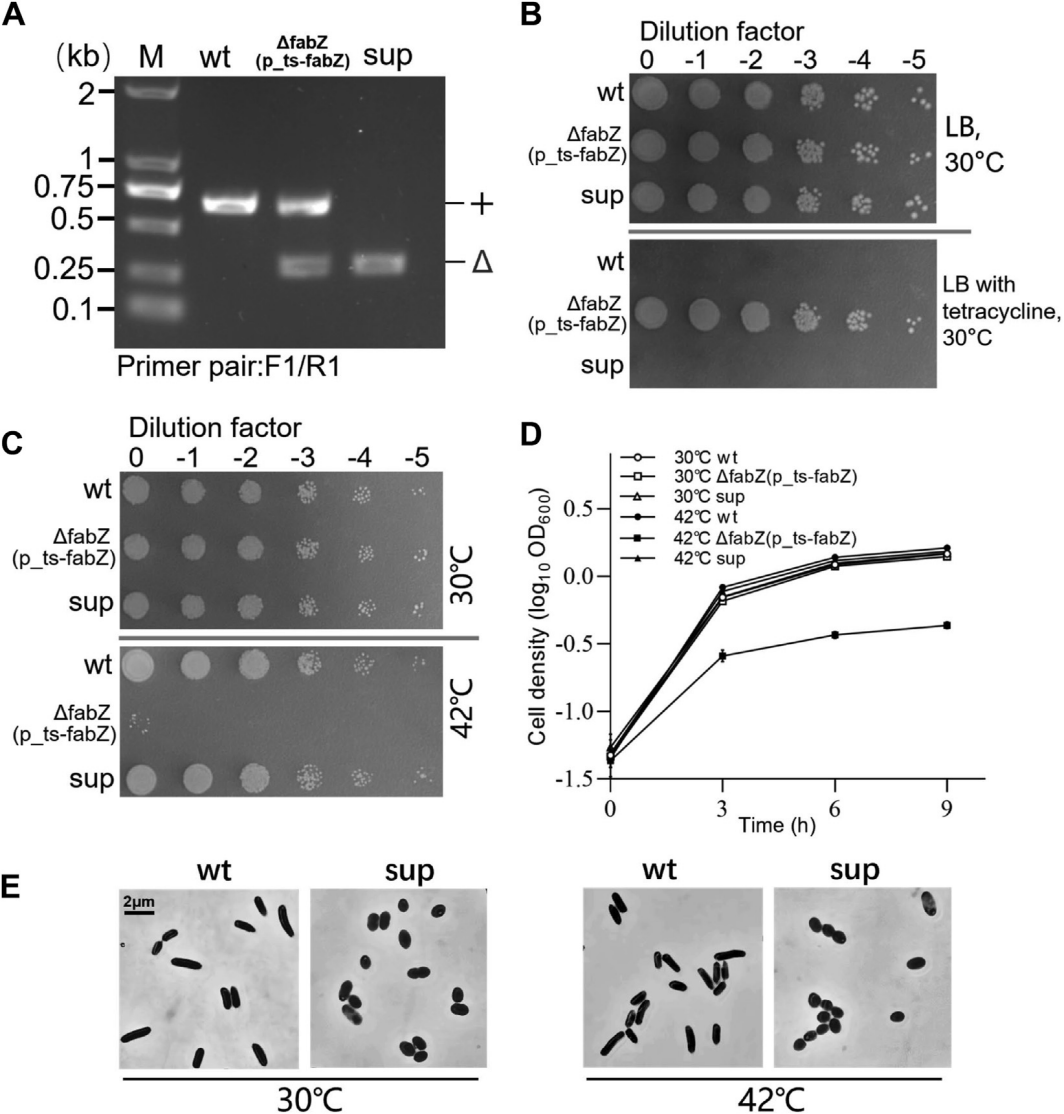
**Isolation and phenotypic analysis of a suppressor of *ΔfabZ*(p_ts-*fabZ*) at a restrictive temperature.***A,* a suppressor (*sup*) is identified from the suppressor screen. A gel image shows the *sup* strain containing only the *ΔfabZ* allele. *B,* spot-plating assay of the WT, *ΔfabZ*(p_ts-*fabZ*), and *sup* treated with or without tetracycline at 30 °C. *C,* spot-plating assay of the WT, *ΔfabZ*(p_ts-*fabZ*), and *sup* at 30 °C and 42 °C. *D,* growth curve analysis of the WT, *ΔfabZ*(p_ts-*fabZ*), and *sup* at 30 °C and 42 °C. *E,* microscopic analysis of the WT and *sup* at 30 °C and 42 °C. ts, temperature-sensitive.

To explore whether the growth defect caused by the *fabZ* deletion could be suppressed in *P. aeruginosa*, we conducted a spontaneous mutagenesis assay as previously described (26, 27). Over 1.0 × 10^9^ *ΔfabZ*(p_ts-*fabZ*) mutant cells were spread on LB plates and incubated at the semirestrictive temperature of 40 °C. After multiple rounds screening, a colony was identified containing only the *ΔfabZ* allele with no WT allele present, as confirmed by PCR analysis (Fig. 5*A*). Furthermore, to exclude cointegrates with the ts plasmid (carrying a tetracycline resistance marker), we confirmed its absence by showing that the suppressor strain was sensitive to tetracycline plates (Fig. 5*B*). This strain was designated as *sup*, representing a suppressor of *ΔfabZ*.

Spot-plating assays and growth curve analyses demonstrated that the *sup* strain successfully rescued the growth defect of *ΔfabZ* at both 30 °C and 42 °C (Fig. 5, *C* and *D*). However, the *sup* mutant still exhibited oval-shaped cell morphology at 30 °C and 42 °C (Fig. 5*E*), resembling the morphology of the *ΔfabZ*(p_ts-*fabZ*) mutant under 42 °C (Fig. 2*B*). These observations suggest that although the *sup* mutant was able to rescue the lethality of *ΔfabZ*, it failed to revert the oval-shaped morphology of *ΔfabZ* cells back to the WT rod-shaped morphology.

### Deletion of *lpxH* identified as a suppressor factor of *ΔfabZ* lethality

**Figure 6:**
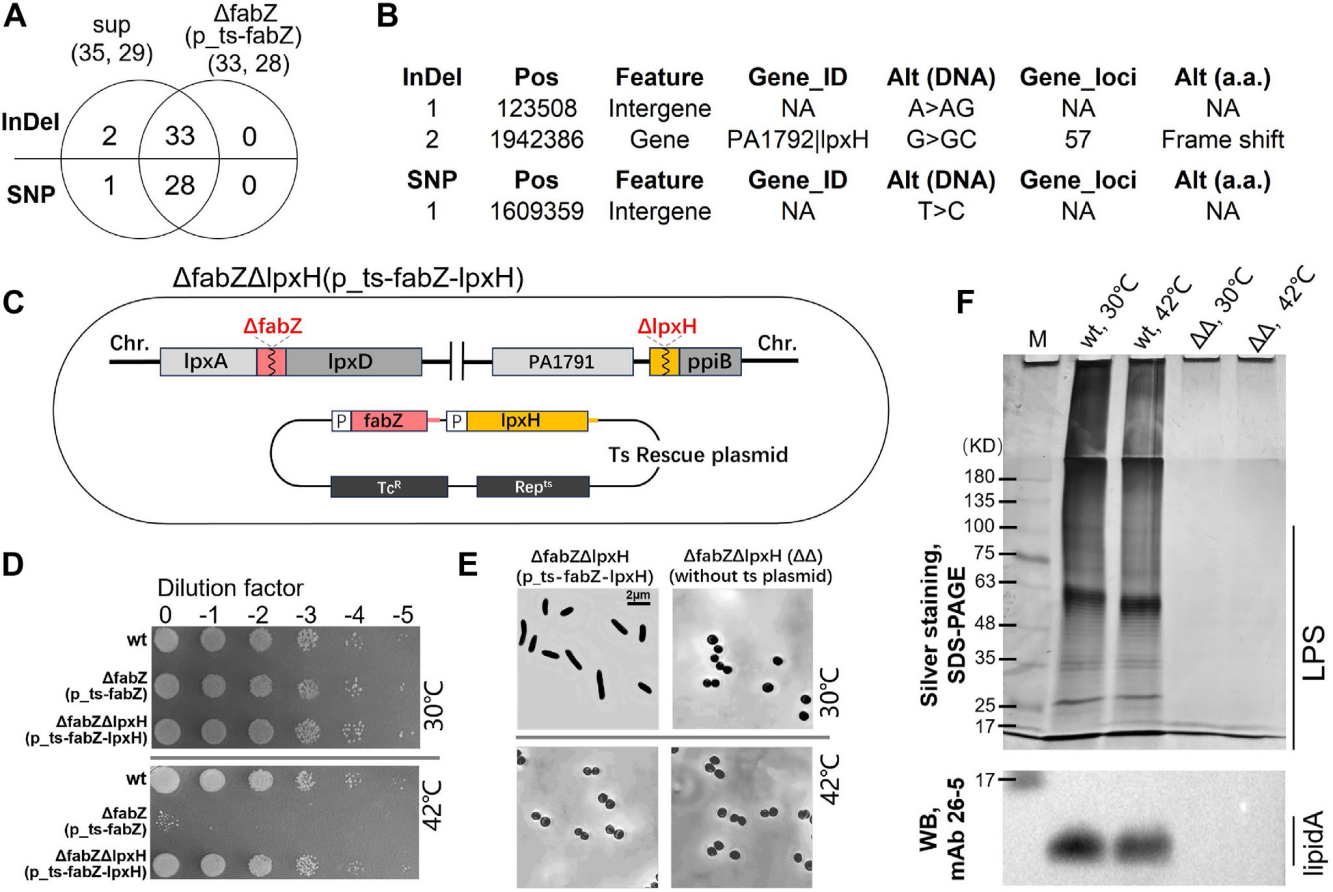
**Deletion of *lpxH*, a candidate suppressor gene identified in *sup*, can suppress the lethal phenotype of *ΔfabZ*.***A,* small mutations of SNP and indel loci detected using genome resequencing methodology; Venn diagram showing the mutation loci observed in *sup* and *ΔfabZ*(p_ts-*fabZ*). *B,* locations of two sup-specific Indel loci and 1 SNP loci. SNP chromosome coordinates (Pos) and nucleotide alteration (Alt) are shown. *C,* schematic map of *ΔfabZΔlpxH*(p_ts-*fabZ-lpxH*). *ΔfabZΔlpxH*(p_ts-*fabZ-lpxH*) contains two deletion allele, *ΔfabZ* and *ΔlpxH*, on the chromosome and two native promoter-controlled complementary copies, *fabZ* and *lpxH*, on the ts rescue plasmid. *D,* spot-plating assay of the *ΔfabZΔlpxH*(p_ts-*fabZ-lpxH*) at 30 °C and 42 °C. *E,* microscopic analysis of *ΔfabZΔlpxH*(p_ts-*fabZ-lpxH*) and *ΔfabZΔlpxH* at 30 °C and 42 °C. *F,* silver-stained SDS-PAGE gel and Western blot image. ΔΔ indicate *ΔfabZΔlpxH.* LPS components are separated on an 8% SDS-PAGE gel (*up panel*). Molecules derived from the *bottom* 8% SDS-PAGE gel are probed with mAb 26-5 against lipid A (*down panel*). ts, temperature-sensitive; LPS, lipopolysaccharide.

To identify candidate suppressor genes in the *sup* mutant compared to *ΔfabZ*(p_ts-*fabZ*), genome resequencing was performed using Illumina technology (see Experimental procedures). Approximately 5 million 300 bp short reads were generated for both *sup* and *ΔfabZ*(p_ts-*fabZ*), with an average sequencing depth of ∼200 × . The reads were aligned to the reference genome (http://www.pseudomonas.com) using Burrows-Wheeler Aligner software (http://bio-bwa.sourceforge.net) (34), and mutations were identified using SAMtools (35). In total, 64 small mutations were identified in the *sup* strain, comprising 29 SNP loci and 35 indel loci (Fig. 6*A*, Table S1). Of these, 95.3% (61 loci) were already present in *ΔfabZ*(p_ts-*fabZ*) (Fig. 6*A*, Table S1), suggesting that these mutations were not responsible for the suppression of *ΔfabZ*.

Among the three sup-specific mutations, one SNP locus and one of the two indel loci were located in intergenic regions, while the other indel was located within the coding sequence of PA1792|*lpxH* (position +57), resulting in a frameshift mutation and subsequent loss of *lpxH* function (Fig. 6*B*). This finding suggested that the loss of *lpxH* function could suppress the lethality of *ΔfabZ*.

To test this hypothesis, we constructed a *ΔfabZΔlpxH*(p_ts-*fabZ-lpxH*) strain by deleting both *fabZ* and *lpxH* on the chromosome while providing complementary copies under their native promoters on a ts rescue plasmid (Figs. 6*C*, S2, *A* and *B*). Unlike the *ΔfabZΔlpxA*(p_ts-*fabZ*-*lpxA*) and *ΔfabZΔlpxC*(p_ts-*fabZ-lpxC*) strains, the *ΔfabZΔlpxH*(p_ts-*fabZ-lpxH*) strain was able to grow at 42 °C (Fig. 6*D*), demonstrating that mutation in *lpxH*, but not *lpxA* or *lpxC*, can rescue the lethality caused by *ΔfabZ* in *P. aeruginosa*.

To further validate this rescue effect, we cultured the *ΔfabZΔlpxH*(p_ts-*fabZ-lpxH*) strain on solid LB medium at 42 °C using the streak plate method. At this restrictive temperature, the ts rescue plasmid is unable to replicate and is consequently lost. Following incubation, individual colonies were screened by PCR to confirm the absence of the ts rescue plasmid, resulting in the isolation of a *ΔfabZΔlpxH* strain (Fig. S2C). Growth curve analyses demonstrated that the *ΔfabZΔlpxH* was able to grow normally at both 30 °C and 42 °C (Fig. S2*D*), further confirming that the loss of *lpxH* rescues the growth defect caused by *ΔfabZ*. Microscopic analysis revealed that the *ΔfabZΔlpxH* mutant exhibited a consistent oval-shaped morphology at both 30 °C and 42 °C, while the *ΔfabZΔlpxH*(p_ts-*fabZ-lpxH*) strain displayed oval-shaped morphology at 42 °C but retained the WT rod-shaped morphology at 30 °C due to the presence of the ts rescue plasmid, which remains stable at permissive temperature of 30 °C (Fig. 6*E*).

We next examined whether lipid A biosynthesis was restored in the *ΔfabZΔlpxH* strain. Western blot analysis demonstrated that lipid A were undetectable in *ΔfabZΔlpxH* strains at both 30 °C and 42 °C (Fig. 6*F*, bottom panel). Similar to the *ΔfabZΔlpxA*(p_ts-*fabZ*-*lpxA*) and *ΔfabZΔlpxC*(p_ts-*fabZ-lpxC*) mutants at 42 °C, silver staining of LPS samples from the *ΔfabZΔlpxH* strain showed no signals (Fig. 6*F*, top panel). These findings suggest that the suppression of *ΔfabZ* lethality by *lpxH* deletion may not be due to a rebalancing of lipid A and phospholipid synthesis, but rather involve alternative mechanisms that stabilize the membrane structure.

### Pentadecanoic acid and heptadecylic acid supplementation rescue the growth defect of *ΔfabZ*(p_ts-*fabZ*) at a restrictive temperature

**Figure 7:**
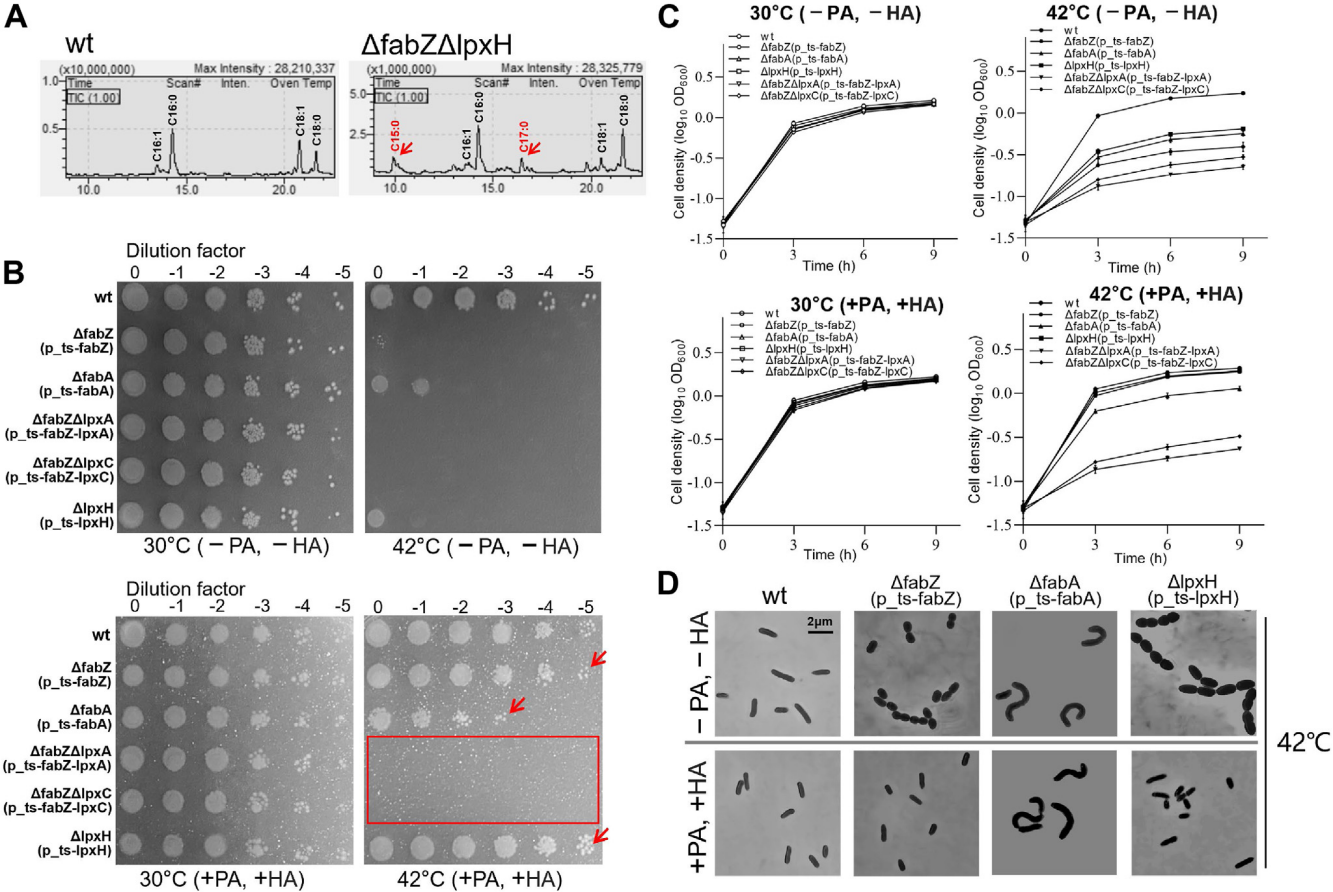
**Supplementation with pentadecanoic acid and heptadecylic acid rescue the growth defect of *ΔfabZ*(p_ts-*fabZ*) at a restrictive temperature.***A,* GC chromatograms of FAME in *ΔfabZΔlpxH* and WT. *B,* the spot-plating assay shows various strains growing on a plate with 0.2% pentadecanoic acid (PA, C15:0) and heptadecanoic acid (HA, C17:0) supplementation. *C,* growth curve analysis of various strains indicated in (B). *D,* microscopic analysis of *ΔfabZ(p_ts-fabZ)*, *ΔfabA*(p_ts-*fabA*), *ΔlpxH*(p_ts-*lpxH*), and WT supplemented with or without PA and HA at 42 °C. ts, temperature-sensitive; FAME, fatty acid methyl ester.

To investigate the changes in fatty acid composition in the *ΔfabZΔlpxH* mutant, we analyzed its fatty acid profile using GC-MS after fatty acid methyl ester (FAME) derivatization (see Experimental procedures). The results revealed that, in addition to the predominant even-chain fatty acids (C16 and C18) observed in the WT strain (27), the *ΔfabZΔlpxH* mutant exhibited significantly increased levels of odd-chain fatty acids, pentadecanoic acid (PA) (C15:0), and heptadecanoic acid (HA) (C17:0) (Figs. 7*A*, S3). To evaluate whether these odd-chain fatty acids could rescue the lethal phenotype of the *ΔfabZ*(p_ts-*fabZ*) at 42 °C, we supplemented the growth medium with 0.2% PA and HA. Spot-plating assays and growth curve analyses demonstrated that the supplementation of PA and HA not only fully restored the growth of *ΔfabZ*(p_ts-*fabZ*) at 42 °C but also completely rescued the growth defect of *ΔlpxH*(p_ts-*lpxH*) (29) and partially rescued *ΔfabA*(p_ts-*fabA*) (27) at the same temperature (Fig. 7, *B* and *C*, see arrows). However, supplementation of PA and HA failed to restore the growth of *ΔfabZΔlpxA*(p_ts-*fabZ*-*lpxA*) and *ΔfabZΔlpxC*(p_ts-*fabZ*-*lpxC*) at 42 °C (Fig. 7, *B* and *C*, see rectangle). Microscopic analysis revealed that the addition of PA and HA also restored the oval-shaped cell morphology of *ΔfabZ*(p_ts-*fabZ*) and *ΔlpxH*(p_ts-*lpxH*) mutants to the WT rod-shaped morphology at 42 °C (Fig. 7*D*). However, this supplementation was unable to revert the curved morphology of *ΔfabA*(p_ts-*fabA*) at 42 °C to the WT rod shape (Fig. 7*D*). These findings suggest that the increased levels of odd-chain fatty acids, such as PA and HA, may play a critical role in stabilizing the membrane and rescuing the lethal phenotype of *ΔfabZ* and *ΔlpxH*.

## Discussion

The *fabZ* gene, a key component of the FASII cycle, encodes an enzyme essential for bacterial fatty acid synthesis, a process critical for maintaining cell membrane integrity and proper cell shape. Disruption of the FASII cycle, whether through enzymatic inhibition or genetic mutations, can result in significant alterations in bacterial morphology, including changes in cell size and shape (27, 36, 37). In this study, we constructed a ts mutant of *fabZ*, *ΔfabZ*(p_ts-*fabZ*), using the previously described three-step method (26, 27, 28, 29) and demonstrated that depletion of *fabZ* in *P. aeruginosa* causes cells to adopt an oval morphology, highlighting its critical role in maintaining the structural integrity of the cell membrane, which is essential for proper cell wall synthesis and bacterial morphology in *P. aeruginosa* (Fig. 2). This observation aligns with findings by Yao *et al.* (37), who reported that loss of FabH, another enzyme in the FASII cycle, leads to a significant reduction in cell size in *E. coli*. However, the morphological changes observed in *fabZ*-depleted cells differ from the curved morphology seen in *fabA*-depleted cells in *P. aeruginosa* (27), emphasizing the essential yet nonredundant roles of *fabZ* and *fabA* in fatty acid biosynthesis and their differential impacts on cell structure.

FabZ and FabA are both β-hydroxyacyl-ACP dehydratases essential for bacterial fatty acid biosynthesis, but they exhibit distinct substrate specificities and functional roles critical for maintaining cellular functions (13, 38). While FabZ facilitates the elongation of both saturated and unsaturated fatty acids with broad substrate specificity, FabA specializes in unsaturated fatty acid synthesis due to its isomerase activity that converts *trans-*2-decenoyl-ACP to *cis-*3-decenoyl-ACP (13, 14). Our findings demonstrate that overexpression of *fabA* cannot rescue the growth defect caused by *fabZ* depletion (Fig. 3*A*), highlighting the nonredundant roles of these enzymes in *P. aeruginosa*. This functional specificity is further emphasized by the inability of oleic or SA supplementation, which rescues *fabA* mutations (27), to restore the viability of *fabZ* mutants (Fig. 3*B*). The inability of *fabA* to replace *fabZ*’s function is likely due to *fabZ*’s broader substrate specificity and its distinct structural characteristics, which are critical for its enzymatic activity and its role in fatty acid elongation (14, 39, 40). While FabA exhibits limited activity toward long-chain unsaturated β-hydroxyacyl-ACPs, FabZ effectively dehydrates both saturated and unsaturated forms, making it indispensable for bacterial fatty acid biosynthesis (22, 41, 42). These results demonstrate the distinct enzymatic functions of FabA and FabZ and their essential yet nonoverlapping roles in fatty acid metabolism in *P. aeruginosa*.

The biosynthesis of lipid A, also known as the Raetz pathway, involves nine essential enzymes required for the synthesis of Kdo_2_-lipid A in *E. coli* (19, 43). The first six enzymes—LpxA, LpxC, LpxD, LpxH, LpxB, and LpxK—are essential for the formation of lipid IV_A_, the minimal structure necessary for the viability of many Gram-negative bacteria (19, 43). Previous studies in *E. coli* and *K. pneumoniae* have demonstrated that mutations in *fabZ* can suppress the growth defects of *lpxA* or *lpxC* mutants, likely through a rebalancing of lipid A and phospholipid biosynthesis (18, 22, 33). However, our study shows that such suppression does not occur in *P. aeruginosa*. Neither *ΔfabZΔlpxA*(p_ts-*fabZ*-*lpxA*) nor *ΔfabZΔlpxC*(p_ts-*fabZ-lpxC*) mutants were viable at a restrictive temperature (Fig. 4), suggesting species-specific differences in lipid metabolism and genetic interactions.

By using the ts plasmid-based mutant strains, we previously identified that overexpression of the *fbp* gene can suppress the growth defect of the *gmhB*(p_ts-*gmhB*) mutant at a restrictive temperature (26), and overexpression of the *desA* gene can suppress the growth defect of the *ΔfabA*(p_ts-*fabA*) mutant under same condition in *P. aeruginosa* (27). In this study, employing the same approach, we identified an insertion mutation within the coding sequence of *lpxH* in the *sup* mutant, resulting in a frameshift mutation, which rescued the lethal phenotype of *ΔfabZ* (Figs. 5 and 6). Further analysis revealed that the *ΔfabZΔlpxH* (without ts plasmid) was viable and exhibited increased levels of odd-chain fatty acids with carbon chain lengths of C15 and C17, as identified by GC-MS after FAME derivatization (Fig. 7*A*). These odd-chain fatty acids likely contribute to membrane stabilization (44, 45), possibly by modulating fluidity and enhancing resistance to oxidative stress. Their accumulation in the *ΔfabZΔlpxH* mutant may help compensate for lipid biosynthesis defects, supporting membrane integrity and cell survival. Supplementation with PA (C15:0) and HA (C17:0), corresponding to the carbon chain lengths identified in the *ΔfabZΔlpxH* mutant through FAME and GC-MS analysis, restored the growth and morphology of *ΔfabZ*(p_ts-*fabZ*) and *ΔlpxH*(p_ts-*lpxH*) mutants at restrictive temperature (Fig. 7). These findings suggest a previously unrecognized regulatory mechanism linking fatty acid biosynthesis and lipid A biosynthesis. Under the dual mutation pressure of *lpxH* and *fabZ*, *P. aeruginosa* compensates by producing odd-chain fatty acids (C15 and C17), which likely stabilize the membrane and support cell survival. While previous studies emphasized the role of phospholipid-to-lipid A balance in maintaining viability, our results reveal an alternative adaptive strategy involving the accumulation of odd-chain fatty acids. Further studies are needed to elucidate the precise molecular mechanisms by which *ΔlpxH* facilitates the survival of *fabZ*-deficient.

In conclusion, this study provides new insights into the genetic interactions between *fabZ* and lipid A biosynthesis genes in *P. aeruginosa*. The identification of *ΔlpxH* as a suppressor factor of *ΔfabZ* lethality and the role of odd-chain fatty acids in membrane stabilization highlight unique aspects of lipid metabolism in this species and suggest potential targets for antimicrobial strategies. Additionally, the plasmid-based ts allele system used in this study proves to be a powerful tool for investigating essential genes and their interactions, offering a framework for exploring the genetic basis of bacterial physiology.

## Experimental procedures

### Oligonucleotides, plasmids, and bacterial strains

Details of oligonucleotides, plasmids, and bacterial strains used are provided in Table 1. The WT *P. aeruginosa* PAO1 strain (BioSciBio) and its derivative strains were grown in LB medium (10 g tryptone, 10 g NaCl, 5 g yeast extract per liter, pH 7.0) with or without 1.5% agar. Media were supplemented with antibiotics (100 μg/ml ampicillin, 50 μg/ml gentamicin, and 100 μg/ml tetracycline) or chemicals (*e.g.*, 10% sucrose, 0.02% or 0.2% arabinose) as needed. Cultures were incubated at 30 °C or 42 °C, depending on the experimental design. Chemicals were obtained from Sigma-Aldrich and MACKLIN.

**Table 1:** Oligonucleotides, plasmids, and strains used in this study

Name	Sequence (5′-3′)	Usage
Oligonucleotides		
F1 or fabZ-F	TCCCTTCACCGATCTCCACTT	Assay fabZ alleles in chr and ts plasmid
R1 or fabZ-R	CGACTTCAGCAGCTGGAAAAAC	Ditto
F2	GTTTCGGTTTCGCCAACGAGAA	Assay fabZ alleles in chr but not ts plasmid
R2	CAGTACTGGTGCACCAGGGTGTAG	Ditto
lpxA-F	CGATGAACTCGATATCCGGGT	Assay lpxA alleles in chr and ts plasmid
lpxC-F	CGGCAAGAATAAATCCCGAAAT	Assay lpxC alleles in chr and ts plasmid
lpxC-R	AGGATGACCTGGATTACCTGGATA	Ditto
lpxH-F	TGATCACGATCATTCCTTGATGC	Assay lpxH alleles in chr and ts plasmid
lpxH-R	TGGACGTGGTCAACAAGATCAAG	Ditto

Name	Relevant genotype	Reference
Plasmids		
pDel	pUC-Gm^r^-sacB	(26, 27, 28, 29)
p_ts	pUC-Tc^r^-ori^ts^	(26, 27, 28, 29)
pOE	pBBRMCS-5-araC-P_BAD_-Gm^r^	(26, 27, 28, 29)
pDel-fabZ	fabZ Deletion cassette in pDel	This study
p_ts-fabZ	fabZ rescue cassette in p_ts	This study
pDel-fabZ&lpxA	fabZ&lpxA Deletion cassette in pDel	This study
p_ts-fabZ&lpxA	fabZ&lpxA rescue cassette in p_ts	This study
pDel-lpxC	lpxC Deletion cassette in pDel	This study
p_ts-fabZ&lpxC	fabZ&lpxC rescue cassette in p_ts	This study
pDel-lpxH	lpxH Deletion cassette in pDel	(29)
p_ts-fabZ&lpxH	fabZ&lpxH rescue cassette in p_ts	This study
pOE-fabZ	araC-P_BAD_-fabZ in pOE	This study
pOE-fabA	araC-P_BAD_-fabA in pOE	(27)
Strains		
PAO1	WT	(26, 27, 28, 29)
ΔfabZ(p_ts-fabZ)	p_ts-fabZ in ΔfabZ	This study
ΔfabZΔlpxA (p_ts-fabZ-lpxA)	p_ts-fabZ-lpxA in ΔfabZΔlpxA	This study
ΔfabZΔlpxC (p_ts-fabZ-lpxC)	p_ts-fabZ-lpxC in ΔfabZΔlpxC	This study
ΔfabZΔlpxH (p_ts-fabZ-lpxH)	p_ts-fabZ-lpxH in ΔfabZΔlpxH	This study
ΔfabZ(p_ts-fabZ) /pOE-fabZ	pOE-fabZ in ΔfabZ(p_ts-fabZ)	This study
ΔfabZ(p_ts-fabZ)/pOE-fabA	pOE-fabA in ΔfabZ(p_ts-fabZ)	This study
sup	sup ΔfabZ	This study
ΔfabZΔlpxH	ΔlpxH in ΔfabZ	This study

ts, temperature-sensitive.

### Plasmid construction

The deletion plasmid and ts rescue plasmid used in this study were the same as those constructed in our previous research (26, 27, 28, 29). To construct the target gene (*e.g.*, *fabZ*, *lpxA*, *lpxC*, *lpxH*) deletion and rescue plasmids, the deletion cassettes and rescue cassettes of these genes were PCR-amplified and cloned into the corresponding deletion plasmids and rescue plasmids using the ClonExpress II one-step cloning kit (Vazyme). Overexpression plasmids were constructed by cloning the araC-P_BAD_ promoter fragment (30) and the coding sequences of target genes into the pBBR1MCS-5 plasmid (31) using the same cloning kit. All constructed plasmids were validated by sequencing before use.

### Plasmid-based ts-mutant strains construction

We used a three-step protocol developed in our previous studies (26, 27, 28, 29) to construct plasmid-based ts mutant strains such as *ΔfabZ*(p_ts-*fabZ*) and dual-gene mutants like *ΔfabZ*Δ*lpxA*(p_ts-*fabZ*-*lpxA*), *ΔfabZ*Δ*lpxC*(p_ts-*fabZ*-*lpxC*), and *ΔfabZ*Δ*lpxH*(p_ts-*fabZ*-*lpxH*). For single-gene ts mutant, the deletion plasmid, which is nonreplicative in *P. aeruginosa*, was first electroporated into the PAO1 strain, and integrants were isolated *via* single-crossover recombination on gentamicin-containing LB plates. The integrant cells were transformed with the ts rescue plasmid carrying *fabZ* under its native promoter and selected on tetracycline-containing plates. Counterselection using *sacB* allowed for the excision of the integrated plasmid on sucrose-containing plates, generating the chromosomal deletion allele (*e.g.*, *ΔfabZ*). For dual-gene ts mutant, such as *ΔfabZ*Δ*lpxC*(p_ts-*fabZ*-*lpxC*), the construction followed a similar process. Initially, a *fabZ* deletion was generated as described above, but the rescue plasmid carried both the *fabZ* and *lpxC* alleles under their respective native promoters. Following this, the deletion plasmid targeting the second gene (*e.g.*, *lpxC*) was electroporated into the strain harboring the *fabZ* deletion (*ΔfabZ*), and a second round of counterselection was performed to generate the chromosomal deletion of the second gene. The resulting dual-gene ts mutants were confirmed by PCR and tested for ts growth phenotypes.

### Spot-plating assay

The spot-plating assay was employed to evaluate the growth of ts-mutant strains. Briefly, overnight cultures were adjusted to the same absorbance (A_600_), followed by 10-fold serial dilutions. The diluted cultures were transferred onto LB agar plates supplemented with the relevant stress factors using a 48-pin replicator (V&P Scientific, Inc). Plates were incubated at 30 °C (permissive temperature) and 42 °C (restrictive temperature) as required, and growth phenotypes were observed after appropriate incubation periods.

### Fluorescence microscopy

Cell morphology was examined using an Olympus BX53 microscope (Olympus) with phase contrast configuration. Nile red (Cat# 7385-67-3, Sinopharm) was used to stain the cytoplasmic membrane, and 4′,6-diamidino-2-phenylindole (Cat# 28718-90-3, Sinopharm) was used to visualize DNA.

### LPS preparation and silver staining

LPS was extracted following the method of Hitchcock and Brown (46). Briefly, 1 ml of fresh bacterial culture (A_600_ = 0.8) was harvested and pelleted. The cell pellets were resuspended in 250 μl lysis buffer (1.0 M Tris, pH 6.8, 10% glycerol, 2% SDS, 4% β-mercaptoethanol) and boiled at 96 °C for 10 min. Proteinase K (200 μg/ml final concentration) was added, and the samples were incubated at 55 °C for 4 h, followed by heating to 70 °C for 1 h to inactivate the enzyme. LPS samples were prepared with SDS-PAGE loading buffer and analyzed by 8% SDS-PAGE. Gels were stained using the silver staining method of Fomsgaard *et al.* (47) and imaged on a Gel Doc system (Bio-Rad).

### Western immunoblotting analysis

LPS samples resolved by SDS-PAGE were transferred to polyvinylidene difluoride membranes (0.22 μm pore size, Millipore) using wet electroblotting at 230 mA for 1 h. The membranes were probed with monoclonal antibodies targeting lipid A (26-5, Abcam). Secondary horseradish peroxidase-conjugated antibodies (Solarbio) were used for signal visualization with ECL reagent (Millipore). The signals were captured using a Tanon 5200 image analyzer (Tanon).

### Isolation of suppressors

More than 1.0 × 10^9^ *ΔfabZ*(p_ts-*fabZ*) cells were plated onto LB plates and incubated at a semirestrictive temperature (40 °C) for 2 weeks to isolate suppressors through spontaneous mutations, as previously described (26, 27). During incubation, plates were maintained in a humid environment with filtered fresh air to ensure optimal growth conditions. Suppressor colonies were validated by streaking on fresh LB plates and incubating at 42 °C. Primer-specific PCR assays were performed to confirm the presence of the *fabZ* deletion allele.

### Genome resequencing analysis

Genomic DNA was extracted using a genomic DNA extraction kit (TaKaRa Bio, Inc.) and sheared to approximately 400 bp fragments using an S2 instrument (Covaris). Sequencing libraries were prepared with the NEXTflex DNA sequencing kit (Bioo Scientific) according to the manufacturer's protocol. The libraries were sequenced on an Illumina MiSeq PE300 platform (Illumina, Inc), generating ∼5 million clean 300 bp reads for both *ΔfabZ*(p_ts-*fabZ*) and suppressor strains. The sequencing was performed at the BioMedical Institute of Shanghai. Reads were aligned to the reference *P. aeruginosa* PAO1 genome (http://www.pseudomonas.com) using Burrows-Wheeler Aligner software (34). SNPs and small insertions and deletions (indels, <50 bp) were identified using SAMtools (35). Structural variants, such as large insertions, deletions, inversions, and translocations, were analyzed using BreakDancer software (https://github.com/genome/breakdancer). A summary of SNP and indel locations identified in the suppressor mutants is provided in Table S1.

### Lipid extraction and FAME preparation

Cellular total lipids were isolated using a chloroform-methanol mixture (2:1, vol/vol) (48, 49). The organic layer was collected into a new tube and dried under nitrogen gas to remove the solvent. The resulting lipid content was weighed to quantify total lipids and then dissolved in hexane to achieve the desired concentration. To analyze fatty acid composition, a portion of the extracted lipids was subjected to transesterification with methanol to produce FAMEs, following a previously established protocol (50).

### GC-MS analyses

FAME species were analyzed using a gas chromatograph (2010Plus GC system, Shimadzu Co) coupled with a mass spectrometer (Shimadzu QP2020). A Rtx-5MS column (30 m × 0.25 mm [i.d.], 0.25 μm film thickness; Restek Co.) was used with helium (99.999%) as the carrier gas. The injection port was set to 260 °C, and samples were injected in splitless mode with a 1-min purge at 50 ml/min. The oven temperature was programmed from 160 °C, increasing at 2 °C/min to 230 °C, and held for 10 min. The mass spectrometer operated in electron ionization mode (70 eV) with a source temperature of 230 °C. Full-scan mass spectra (50–500 *m/z*) were acquired. Peaks were analyzed with LabSolutions software (https://www.shimadzu.com/an/products/software-informatics/labsolutions-series/labsolutionsgcms/index.html) and identified using the NIST 14 Library.

### Statistical analysis

All growth curve data are expressed as mean ± standard error.

## Data availability

All the data presented in this document can be found within the article and accompanying supplementary files.

## Supporting information

This article contains supporting information.

## Conflicts of interest

The authors declare that they have no conflicts of interest with the contents of this article.

## References

[1] Davies J.C. (2002). Pseudomonas aeruginosa in cystic fibrosis: pathogenesis and persistence. Paediatric Respir. Rev..

[2] Aloush V., Navon-Venezia S., Seigman-Igra Y., Cabili S., Carmeli Y. (2006). Multidrug-resistant Pseudomonas aeruginosa: risk factors and clinical impact. Antimicrob. Agents Chemother..

[3] Horcajada J.P., Montero M., Oliver A., Sorlí L., Luque S., Gómez-Zorrilla S. (2019). Epidemiology and treatment of multidrug-resistant and extensively drug-resistant Pseudomonas aeruginosa infections. Clin. Microbiol. Rev..

[4] Tacconelli E., Carrara E., Savoldi A., Harbarth S., Mendelson M., Monnet D.L. (2018). Discovery, research, and development of new antibiotics: the WHO priority list of antibiotic-resistant bacteria and tuberculosis. Lancet Infect. Dis..

[5] Lee S.A., Gallagher L.A., Thongdee M., Staudinger B.J., Lippman S., Singh P.K. (2015). General and condition-specific essential functions of Pseudomonas aeruginosa. Proc. Natl. Acad. Sci..

[6] Fields F.R., Lee S.W., McConnell M.J. (2017). Using bacterial genomes and essential genes for the development of new antibiotics. Biochem. Pharmacol..

[7] Juhas M., Eberl L., Church G.M. (2012). Essential genes as antimicrobial targets and cornerstones of synthetic biology. Trends Biotechnology.

[8] Heath R.J., Rock C.O. (2004). Fatty acid biosynthesis as a target for novel antibacterials. Curr. Opin. Invest. Drugs.

[9] Zhang Y.-M., White S.W., Rock C.O. (2006). Inhibiting bacterial fatty acid synthesis. J. Biol. Chem..

[10] Yao J., Rock C.O. (2017). Bacterial fatty acid metabolism in modern antibiotic discovery. Biochim. Biophys. Acta Mol. Cell Biol. Lipids.

[11] Heath R.J., White S.W., Rock C.O. (2001). Lipid biosynthesis as a target for antibacterial agents. Prog. Lipid Res..

[12] Wang Y., Ma S. (2013). Recent advances in inhibitors of bacterial fatty acid synthesis type II (FASII) system enzymes as potential antibacterial agents. ChemMedChem.

[13] Heath R.J., Rock C.O. (1996). Roles of the FabA and FabZ β-hydroxyacyl-acyl carrier protein dehydratases in Escherichia coli fatty acid biosynthesis. J. Biol. Chem..

[14] Dodge G.J., Patel A., Jaremko K.L., McCammon J.A., Smith J.L., Burkart M.D. (2019). Structural and dynamical rationale for fatty acid unsaturation in Escherichia coli. Proc. Natl. Acad. Sci..

[15] Charbon G., Riber L., Cohen M., Skovgaard O., Fujimitsu K., Katayama T. (2011). Suppressors of DnaAATP imposed overinitiation in Escherichia coli. Mol. Microbiol..

[16] Van Leeuwen J., Pons C., Tan G., Wang Z.Y., Hou J., Weile J. (2020). Systematic analysis of bypass suppression of essential genes. Mol. Syst. Biol..

[17] Kloser A., Laird M., Deng M., Misra R. (1998). Modulations in lipid A and phospholipid biosynthesis pathways influence outer membrane protein assembly in Escherichia coli K-12. Mol. Microbiol..

[18] Zeng D., Zhao J., Chung H.S., Guan Z., Raetz C.R., Zhou P. (2013). Mutants resistant to LpxC inhibitors by rebalancing cellular homeostasis. J. Biol. Chem..

[19] Raetz C.R., Reynolds C.M., Trent M.S., Bishop R.E. (2007). Lipid A modification systems in gram-negative bacteria. Annu. Rev. Biochem..

[20] Lam J.S., Taylor V.L., Islam S.T., Hao Y., Kocíncová D. (2011). Genetic and functional diversity of Pseudomonas aeruginosa lipopolysaccharide. Front. Microbiol..

[21] Simpson B.W., Trent M.S. (2019). Pushing the envelope: LPS modifications and their consequences. Nat. Rev. Microbiol..

[22] Mohan S., Kelly T.M., Eveland S.S., Raetz C., Anderson M.S. (1994). An Escherichia coli gene (FabZ) encoding (3R)-hydroxymyristoyl acyl carrier protein dehydrase. Relation to fabA and suppression of mutations in lipid A biosynthesis. J. Biol. Chem..

[23] Williams A.H., Raetz C.R. (2007). Structural basis for the acyl chain selectivity and mechanism of UDP-N-acetylglucosamine acyltransferase. Proc. Natl. Acad. Sci..

[24] Zhou P., Barb A.W. (2008). Mechanism and inhibition of LpxC: an essential zinc-dependent deacetylase of bacterial lipid A synthesis. Curr. Pharm. Biotechnol..

[25] Whittington D.A., Rusche K.M., Shin H., Fierke C.A., Christianson D.W. (2003). Crystal structure of LpxC, a zinc-dependent deacetylase essential for endotoxin biosynthesis. Proc. Natl. Acad. Sci..

[26] Yang Z., Zhang Z., Zhu J., Ma Y., Wang J., Liu J. (2022). Analysis of the plasmid-based ts allele of PA0006 reveals its function in regulation of cell morphology and biosynthesis of core lipopolysaccharide in Pseudomonas aeruginosa. Appl. Environ. Microbiol..

[27] Tian L., Yang Z., Wang J., Liu J. (2023). Analysis of the plasmid-based ts-mutant Δ fabA/pTS-fabA reveals its lethality under aerobic growth conditions that is suppressed by mild overexpression of desA at a restrictive temperature in Pseudomonas aeruginosa. Microbiol. Spectr..

[28] Zhu J., Zhao H., Yang Z. (2024). Genetic analysis of the ts-lethal mutant Δpa0665/pTS-pa0665 reveals its role in cell morphology and oxidative phosphorylation in Pseudomonas aeruginosa. Genes.

[29] Zhang H., Yang Z., Liu J. (2024). Genetic analysis of the plasmid-based temperature-lethal mutant pa1792| lpxH (Ts) in Pseudomonas aeruginosa. Genes.

[30] Guzman L.-M., Belin D., Carson M.J., Beckwith J. (1995). Tight regulation, modulation, and high-level expression by vectors containing the arabinose PBAD promoter. J. Bacteriol..

[31] Kovach M.E., Elzer P.H., Hill D.S., Robertson G.T., Farris M.A., Roop II R.M. (1995). Four new derivatives of the broad-host-range cloning vector pBBR1MCS, carrying different antibiotic-resistance cassettes. Gene.

[32] Onishi H.R., Pelak B.A., Gerckens L.S., Silver L.L., Kahan F.M., Chen M.-H. (1996). Antibacterial agents that inhibit lipid A biosynthesis. Science.

[33] Mostafavi M., Wang L., Xie L., Takeoka K.T., Richie D.L., Casey F. (2018). Interplay of Klebsiella pneumoniae fabZ and lpxC mutations leads to LpxC inhibitor-dependent growth resulting from loss of membrane homeostasis. mSphere.

[34] Li H., Durbin R. (2009). Fast and accurate short read alignment with Burrows–Wheeler transform. Bioinformatics.

[35] Li H., Handsaker B., Wysoker A., Fennell T., Ruan J., Homer N. (2009). The sequence alignment/map format and SAMtools. Bioinformatics.

[36] Wachi M., Doi M., Tamaki S., Park W., Nakajima-Iijima S., Matsuhashi M. (1987). Mutant isolation and molecular cloning of mre genes, which determine cell shape, sensitivity to mecillinam, and amount of penicillin-binding proteins in Escherichia coli. J. Bacteriol..

[37] Yao Z., Davis R.M., Kishony R., Kahne D., Ruiz N. (2012). Regulation of cell size in response to nutrient availability by fatty acid biosynthesis in Escherichia coli. Proc. Natl. Acad. Sci..

[38] Zhu L., Cheng J., Luo B., Feng S., Lin J., Wang S. (2009). Functions of the Clostridium acetobutylicium FabF and FabZ proteins in unsaturated fatty acid biosynthesis. BMC Microbiol..

[39] Leesong M., Henderson B.S., Gillig J.R., Schwab J.M., Smith J.L. (1996). Structure of a dehydratase–isomerase from the bacterial pathway for biosynthesis of unsaturated fatty acids: two catalytic activities in one active site. Structure.

[40] Lu Y.-J., White S.W., Rock C.O. (2005). Domain swapping between Enterococcus faecalis FabN and FabZ proteins localizes the structural determinants for isomerase activity. J. Biol. Chem..

[41] Marrakchi H., Choi K.-H., Rock C.O. (2002). A new mechanism for anaerobic unsaturated fatty acid formation inStreptococcus pneumoniae. J. Biol. Chem..

[42] Kimber M.S., Martin F., Lu Y., Houston S., Vedadi M., Dharamsi A. (2004). The structure of (3R)-hydroxyacyl-acyl carrier protein dehydratase (FabZ) from Pseudomonas aeruginosa. J. Biol. Chem..

[43] Dowhan W. (2011). The Raetz pathway for lipid A biosynthesis: Christian Rudolf Hubert Raetz, MD PhD, 1946–2011. J. Lipid Res..

[44] Jenkins B., West J.A., Koulman A. (2015). A review of odd-chain fatty acid metabolism and the role of pentadecanoic acid (C15: 0) and heptadecanoic acid (C17: 0) in health and disease. Molecules.

[45] Venn-Watson S. (2024). The cellular stability hypothesis: evidence of ferroptosis and accelerated aging-associated diseases as newly identified nutritional pentadecanoic acid (C15: 0) deficiency syndrome. Metabolites.

[46] Hitchcock P.J., Brown T.M. (1983). Morphological heterogeneity among Salmonella lipopolysaccharide chemotypes in silver-stained polyacrylamide gels. J. Bacteriol..

[47] Fomsgaard A., Freudenberg M., Galanos C. (1990). Modification of the silver staining technique to detect lipopolysaccharide in polyacrylamide gels. J. Clin. Microbiol..

[48] Hancock I., Meadow P.M. (1969). The extractable lipids of Pseudomonas aeruginosa. Biochim. Biophys. Acta.

[49] Moss C., Dees S. (1976). Cellular fatty acids and metabolic products of Pseudomonas species obtained from clinical specimens. J. Clin. Microbiol..

[50] Van Wijngaarden D. (1967). Modified rapid preparation of fatty acid esters from lipids for gas chromatographic analysis. Anal. Chem..

