# Supplementary material for: Conditional lethality and suppressor analysis of plasmid-based temperature-sensitive fabZ expression in Pseudomonas aeruginosa
Source: J Biol Chem. 2025 Apr 26;301(6):108553. doi: 10.1016/j.jbc.2025.108553 (PMC12152623; doi:10.1016/j.jbc.2025.108553)
Supplement: Table S1 [file mmc1.docx]

| **Supplementary table S1. Small indel and SNP analysis in *sup* and *ΔfabZ*(p_ts-*fabZ*)** | | | | | | | | |  |  |  |  |  |  |  |  |  |
| --- | --- | --- | --- | --- | --- | --- | --- | --- | --- | --- | --- | --- | --- | --- | --- | --- | --- |
|  |  |  |  |  |  |  |  |  |  |  |  |  |  |  |  |  |  |
| **(A) Indel loci specific to sup, not present in *ΔfabZ*(p_ts-*fabZ*)** | | | | | | |  |  |  |  |  |  |  |  |  |  |  |
| **sup** |  |  |  |  |  |  |  |  | ***ΔfabZ***  **(p_ts-*fabZ*)** |  |  |  |  |  |  |  |  |
| **POS_sup** | **REF** | **ALT** | **alt-coverage** | **all-coverage** | **alt-precent** | **Gene_loci** | **Gene_ID** | **function** | **POS_sup** | **REF** | **ALT** | **alt-coverage** | **all-coverage** | **alt-precent** | **Gene_loci** | **Gene_ID** | **function** |
| 123508 | A | AG | 235 | 235 | 1 |  |  |  | NA |  |  |  |  |  |  |  |  |
| 1942386 | G | GC | 216 | 216 | 1 | 57 | PA1792 | conserved hypothetical protein | NA |  |  |  |  |  |  |  |  |
|  |  |  |  |  |  |  |  |  |  |  |  |  |  |  |  |  |  |
| **(B) SNP loci specific to sup, not present in *ΔfabZ*(p_ts-*fabZ*)** | | | | | | |  |  |  |  |  |  |  |  |  |  |  |
| **sup** |  |  |  |  |  |  |  |  | ***ΔfabZ***  **(p_ts-*fabZ*)** |  |  |  |  |  |  |  |  |
| **POS** | **REF** | **ALT** | **alt-coverage** | **all-coverage** | **alt-precent** | **Gene_loci** | **Gene_ID** | **function** | **POS** | **REF** | **ALT** | **alt-coverage** | **all-coverage** | **alt-precent** | **Gene_loci** | **Gene_ID** | **function** |
| 1609359 | T | C | 122 | 122 | 1 |  |  |  | NA |  |  |  |  |  |  |  |  |
|  |  |  |  |  |  |  |  |  |  |  |  |  |  |  |  |  |  |
| **(C) Indel loci in both sup and *ΔfabZ*(p_ts-*fabZ*)** | | | | |  |  |  |  |  |  |  |  |  |  |  |  |  |
| **sup** |  |  |  |  |  |  |  |  | ***ΔfabZ***  **(p_ts-*fabZ*)** |  |  |  |  |  |  |  |  |
| **POS_sup** | **REF** | **ALT** | **alt-coverage** | **all-coverage** | **alt-precent** | **Gene_loci** | **Gene_ID** | **function** | **POS** | **REF** | **ALT** | **alt-coverage** | **all-coverage** | **alt-precent** | **Gene_loci** | **Gene_ID** | **function** |
| 169283 | CG | C | 232 | 232 | 1 |  |  |  | 169283 | CG | C | 227 | 236 | 0.96 |  |  |  |
| 411125 | AC | A | 226 | 227 | 1 |  |  |  | 411125 | AC | A | 247 | 247 | 1 |  |  |  |
| 667028 | G | GC | 248 | 250 | 0.99 |  |  |  | 667028 | G | GC | 237 | 237 | 1 |  |  |  |
| 740419 | G | GC | 200 | 200 | 1 | 217 | PA0683 | probable type II secretion system protein | 740419 | G | GC | 171 | 171 | 1 | 217 | PA0683 | probable type II secretion system protein |
| 816529 | G | GC | 170 | 171 | 0.99 |  |  |  | 816529 | G | GC | 153 | 156 | 0.98 |  |  |  |
| 891099 | A | AC | 227 | 232 | 0.98 |  |  |  | 891099 | A | AC | 194 | 204 | 0.95 |  |  |  |
| 1116213 | G | GC | 400 | 403 | 0.99 | 1 | PA1029 | hypothetical protein | 1116213 | G | GC | 335 | 335 | 1 | 1 | PA1029 | hypothetical protein |
| 1215657 | A | AG | 195 | 195 | 1 | 374 | PA1122 | probable peptide deformylase | 1215657 | A | AG | 216 | 216 | 1 | 374 | PA1122 | probable peptide deformylase |
| 1275766 | GA | G | 251 | 253 | 0.99 | 33 | PA1174 | periplasmic nitrate reductase protein NapA | 1275766 | GA | G | 277 | 284 | 0.98 | 33 | PA1174 | periplasmic nitrate reductase protein NapA |
| 1440622 | CA | C | 222 | 224 | 0.99 | 1917 | PA1327 | probable protease | 1440622 | CA | C | 212 | 212 | 1 | 1917 | PA1327 | probable protease |
| 1445357 | A | AG | 252 | 252 | 1 |  |  |  | 1445357 | A | AG | 239 | 239 | 1 |  |  |  |
| 1467482 | A | AG | 252 | 252 | 1 |  |  |  | 1467482 | A | AG | 263 | 265 | 0.99 |  |  |  |
| 1467483 | C | CG | 253 | 253 | 1 |  |  |  | 1467483 | C | CG | 257 | 259 | 0.99 |  |  |  |
| 1835045 | G | GC | 192 | 192 | 1 | 653 | PA1685 | enolase-phosphatase E-1 | 1835045 | G | GC | 222 | 222 | 1 | 653 | PA1685 | enolase-phosphatase E-1 |
| 2169348 | A | AG | 248 | 251 | 0.99 |  |  |  | 2169348 | A | AG | 207 | 208 | 1 |  |  |  |
| 2186927 | G | GC | 189 | 206 | 0.92 |  |  |  | 2186927 | G | GC | 172 | 172 | 1 |  |  |  |
| 2195457 | G | GC | 111 | 111 | 1 |  |  |  | 2195457 | G | GC | 145 | 145 | 1 |  |  |  |
| 2239555 | A | AG | 147 | 147 | 1 |  |  |  | 2239555 | A | AG | 159 | 159 | 1 |  |  |  |
| 2342110 | C | CT | 338 | 338 | 1 |  |  |  | 2342110 | C | CT | 372 | 372 | 1 |  |  |  |
| 2355771 | A | AG | 260 | 260 | 1 | 88 | PA2139 | hypothetical protein | 2355771 | A | AG | 215 | 215 | 1 | 88 | PA2139 | hypothetical protein |
| 2356681 | GC | G | 185 | 185 | 1 | 514 | PA2141 | hypothetical protein | 2356681 | GC | G | 210 | 210 | 1 | 514 | PA2141 | hypothetical protein |
| 2532046 | G | GC | 137 | 137 | 1 |  |  |  | 2532046 | G | GC | 136 | 136 | 1 |  |  |  |
| 2753522 | G | GC | 143 | 143 | 1 | 924 | PA2452 | hypothetical protein | 2753522 | G | GC | 167 | 167 | 1 | 924 | PA2452 | hypothetical protein |
| 2807693 | TCGGCCAGC | T | 216 | 216 | 1 | 225 | PA2492 | transcriptional regulator MexT | 2807693 | TCGGCCAGC | T | 226 | 226 | 1 | 225 | PA2492 | transcriptional regulator MexT |
| 3016844 | G | GC | 389 | 389 | 1 | 41 | PA2668 | hypothetical protein | 3016844 | G | GC | 394 | 394 | 1 | 41 | PA2668 | hypothetical protein |
| 3083196 | A | AG | 168 | 168 | 1 | 3347 | PA2727 | hypothetical protein | 3083196 | A | AG | 142 | 142 | 1 | 3347 | PA2727 | hypothetical protein |
| 3919508 | G | GC | 308 | 309 | 1 |  |  |  | 3919508 | G | GC | 284 | 284 | 1 |  |  |  |
| 4539468 | G | GC | 174 | 178 | 0.98 | 313 | PA4059 | hypothetical protein | 4539468 | G | GC | 155 | 155 | 1 | 313 | PA4059 | hypothetical protein |
| 4888194 | A | AG | 244 | 244 | 1 | 2 | PA4360 | hypothetical protein | 4888194 | A | AG | 199 | 201 | 0.99 | 2 | PA4360 | hypothetical protein |
| 5033101 | G | GC | 214 | 214 | 1 | 1614 | PA4496 | probable binding protein component of ABC transporter | 5033101 | G | GC | 192 | 192 | 1 | 1614 | PA4496 | probable binding protein component of ABC transporter |
| 5071543 | AACTG | A | 417 | 417 | 1 |  |  |  | 5071543 | AACTG | A | 465 | 465 | 1 |  |  |  |
| 5472415 | C | CG | 237 | 237 | 1 |  |  |  | 5472415 | C | CG | 203 | 203 | 1 |  |  |  |
| 5655220 | C | CCGG | 172 | 175 | 0.98 | 410 | PA5024 | conserved hypothetical protein | 5655220 | C | CCGG | 190 | 190 | 1 | 410 | PA5024 | conserved hypothetical protein |
|  |  |  |  |  |  |  |  |  |  |  |  |  |  |  |  |  |  |
| **(D) SNP loci in both sup and *ΔfabZ*(p_ts-*fabZ*)** | | | | |  |  |  |  |  |  |  |  |  |  |  |  |  |
| **sup** |  |  |  |  |  |  |  |  | ***ΔfabZ***  **(p_ts-*fabZ*)** |  |  |  |  |  |  |  |  |
| **POS** | **REF** | **ALT** | **alt-coverage** | **all-coverage** | **alt-precent** | **Gene_loci** | **Gene_ID** | **function** | **POS** | **REF** | **ALT** | **alt-coverage** | **all-coverage** | **alt-precent** | **Gene_loci** | **Gene_ID** | **function** |
| 183697 | T | G | 585 | 586 | 1 | 930 | PA0159 | probable transcriptional regulator | 183697 | T | G | 542 | 542 | 1 | 930 | PA0159 | probable transcriptional regulator |
| 413850 | T | C | 291 | 291 | 1 |  |  |  | 413850 | T | C | 243 | 243 | 1 |  |  |  |
| 721611 | C | T | 292 | 292 | 1 |  |  |  | 721611 | C | T | 212 | 213 | 1 |  |  |  |
| 721622 | C | T | 278 | 278 | 1 |  |  |  | 721622 | C | T | 203 | 203 | 1 |  |  |  |
| 721663 | T | C | 163 | 165 | 0.99 |  |  |  | 721663 | T | C | 118 | 118 | 1 |  |  |  |
| 721664 | C | T | 157 | 159 | 0.99 |  |  |  | 721664 | C | T | 115 | 115 | 1 |  |  |  |
| 721667 | A | G | 139 | 141 | 0.99 |  |  |  | 721667 | A | G | 106 | 106 | 1 |  |  |  |
| 721670 | A | G | 128 | 130 | 0.98 |  |  |  | 721670 | A | G | 91 | 91 | 1 |  |  |  |
| 721718 | A | G | 110 | 111 | 0.99 |  |  |  | 721718 | A | G | 66 | 66 | 1 |  |  |  |
| 721725 | C | T | 106 | 107 | 0.99 |  |  |  | 721725 | C | T | 71 | 71 | 1 |  |  |  |
| 721740 | C | T | 103 | 103 | 1 |  |  |  | 721740 | C | T | 67 | 67 | 1 |  |  |  |
| 1589438 | G | C | 332 | 332 | 1 | 101 | PA1459 | probable methyltransferase | 1589438 | G | C | 351 | 351 | 1 | 101 | PA1459 | probable methyltransferase |
| 2239547 | T | G | 146 | 146 | 1 |  |  |  | 2239547 | T | G | 162 | 162 | 1 |  |  |  |
| 2669175 | G | C | 208 | 208 | 1 | 2455 | PA2400 | PvdJ | 2669175 | G | C | 211 | 211 | 1 | 2455 | PA2400 | PvdJ |
| 2807982 | T | A | 204 | 204 | 1 | 514 | PA2492 | transcriptional regulator MexT | 2807982 | T | A | 192 | 192 | 1 | 514 | PA2492 | transcriptional regulator MexT |
| 4212201 | A | G | 191 | 191 | 1 | 1907 | PA3760 | N-Acetyl-D-Glucosamine phosphotransferase system transporter | 4212201 | A | G | 220 | 220 | 1 | 1907 | PA3760 | N-Acetyl-D-Glucosamine phosphotransferase system transporter |
| 4344266 | A | G | 268 | 268 | 1 | 570 | PA3877 | nitrite extrusion protein 1 | 4344266 | A | G | 209 | 213 | 0.98 | 570 | PA3877 | nitrite extrusion protein 1 |
| 4448855 | C | G | 155 | 167 | 0.93 |  |  |  | 4448855 | C | G | 246 | 259 | 0.95 |  |  |  |
| 4448856 | G | C | 165 | 171 | 0.96 |  |  |  | 4448856 | G | C | 266 | 266 | 1 |  |  |  |
| 4869855 | T | G | 184 | 184 | 1 | 474 | PA4341 | probable transcriptional regulator | 4869855 | T | G | 146 | 146 | 1 | 474 | PA4341 | probable transcriptional regulator |
| 4924552 | C | G | 315 | 315 | 1 | 532 | PA4394 | conserved hypothetical protein | 4924552 | C | G | 331 | 331 | 1 | 532 | PA4394 | conserved hypothetical protein |
| 4924553 | G | C | 312 | 312 | 1 | 531 | PA4394 | conserved hypothetical protein | 4924553 | G | C | 331 | 331 | 1 | 531 | PA4394 | conserved hypothetical protein |
| 5036891 | A | C | 423 | 429 | 0.99 |  |  |  | 5036891 | A | C | 416 | 416 | 1 |  |  |  |
| 5743461 | C | G | 203 | 203 | 1 | 1293 | PA5100 | urocanase | 5743461 | C | G | 224 | 224 | 1 | 1293 | PA5100 | urocanase |
| 5743462 | G | C | 204 | 204 | 1 | 1292 | PA5100 | urocanase | 5743462 | G | C | 222 | 222 | 1 | 1292 | PA5100 | urocanase |
| 6079222 | A | G | 256 | 256 | 1 | 1179 | PA5399 | DgcB, Dimethylglycine catabolism | 6079222 | A | G | 231 | 231 | 1 | 1179 | PA5399 | DgcB, Dimethylglycine catabolism |
| 6098781 | G | C | 143 | 143 | 1 | 1758 | PA5418 | sarcosine oxidase alpha subunit | 6098781 | G | C | 179 | 179 | 1 | 1758 | PA5418 | sarcosine oxidase alpha subunit |
| 6115455 | T | G | 278 | 278 | 1 | 858 | PA5434 | tryptophan permease | 6115455 | T | G | 293 | 293 | 1 | 858 | PA5434 | tryptophan permease |
