# Supplementary material for: Conditional lethality and suppressor analysis of plasmid-based temperature-sensitive fabZ expression in Pseudomonas aeruginosa
Source: J Biol Chem. 2025 Apr 26;301(6):108553. doi: 10.1016/j.jbc.2025.108553 (PMC12152623; doi:10.1016/j.jbc.2025.108553)
Supplement: Figure S1 [file mmc2.pdf]

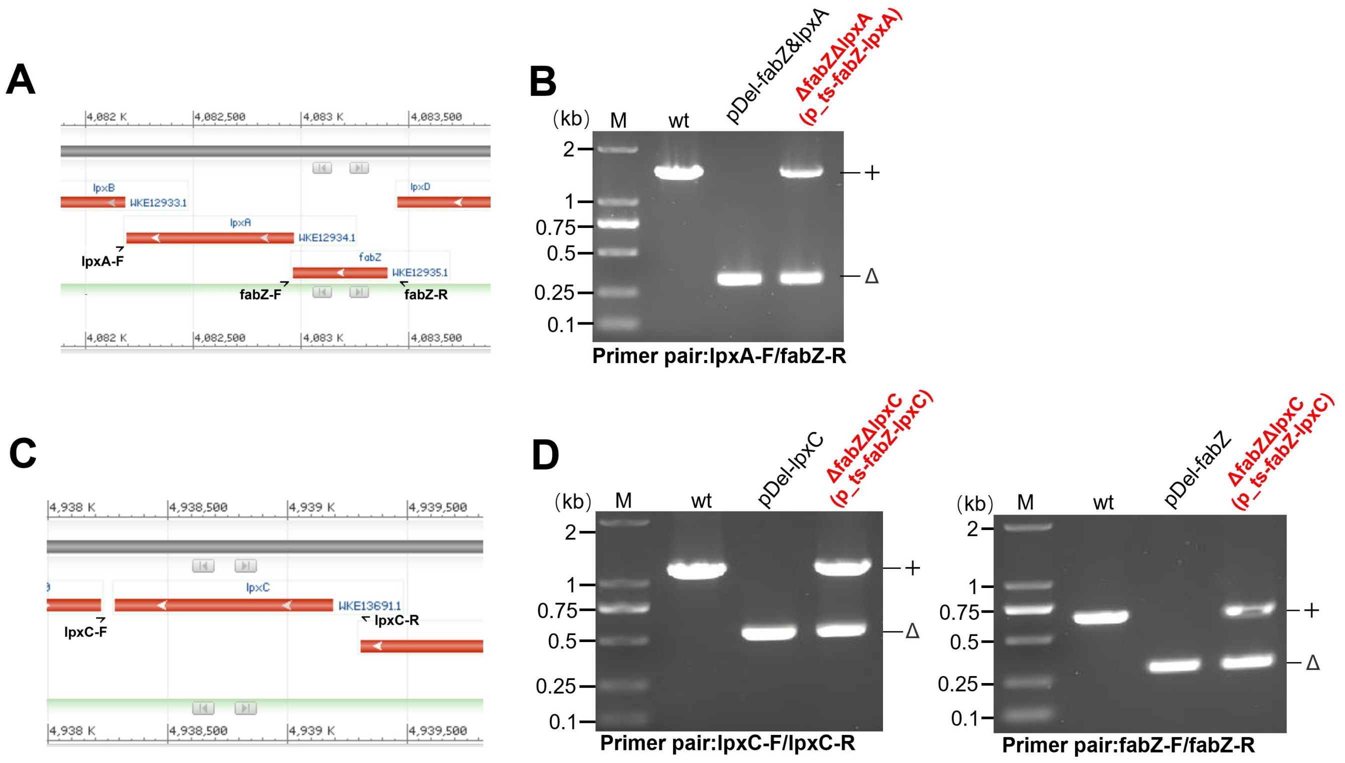

Supplementary Figure S1. PCR identification of *ΔfabZΔlpxA*(p_ts-*fabZ-lpxA*) and *ΔfabZΔlpxC*(p_ts-*fabZ-lpxC*) using gene-specific primer pairs. (A) Schematic representation of primer locations used for *fabZ* and *lpxA* verification. Primers specific to *fabZ* and *lpxA* were designed to distinguish between the wild-type allele and the deletion allele. (B) PCR results for *ΔfabZΔlpxA*(p_ts-*fabZ-lpxA*). Due to the presence of the ts rescue plasmid, PCR products from both the wild-type allele (labeled as "+") and the deletion allele (labeled as "Δ") are observed. The PCR fragment derived from the deletion allele is significantly smaller than that from the wild-type allele. (C) Schematic representation of primer locations used for *lpxC* verification. (D) PCR identification results for *ΔfabZΔlpxC*(p_ts-*fabZ-lpxC*).
