# Supplementary material for: Conditional lethality and suppressor analysis of plasmid-based temperature-sensitive fabZ expression in Pseudomonas aeruginosa
Source: J Biol Chem. 2025 Apr 26;301(6):108553. doi: 10.1016/j.jbc.2025.108553 (PMC12152623; doi:10.1016/j.jbc.2025.108553)
Supplement: Figure S2 [file mmc3.pdf]

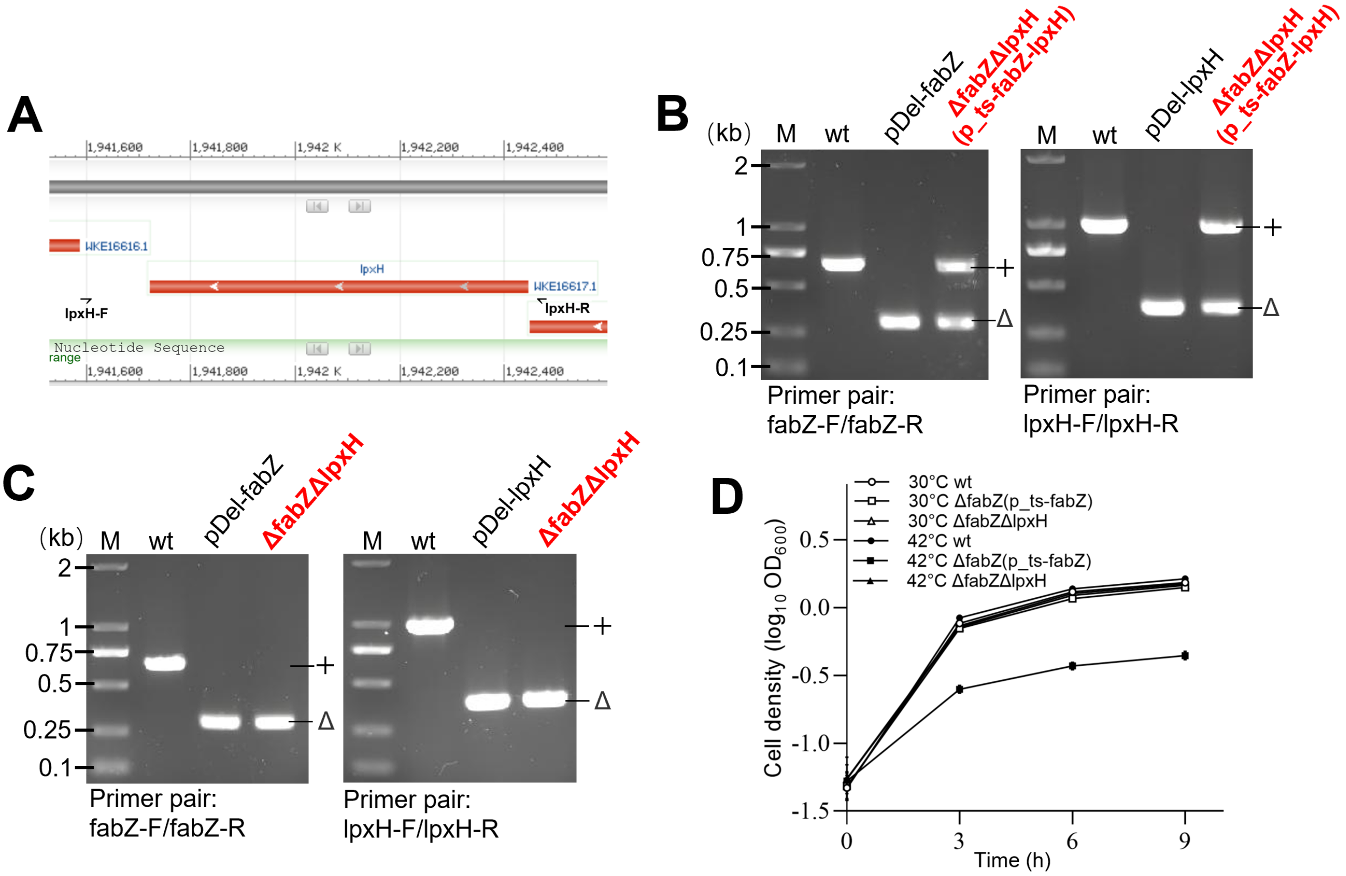

Supplementary Figure S2. PCR identification and growth curve analysis of *ΔfabZΔlpxH*. (A) Schematic representation of primer locations used for *lpxH* verification. Primers specific to *lpxH* were designed to distinguish between the wild-type allele and the deletion allele. (B) PCR identification results for *ΔfabZΔlpxH*(p_ts-*fabZ-lpxH*). (C) PCR identification results for *ΔfabZΔlpxH.* (D) Growth curve analysis of the wild-type, *ΔfabZ*(p_ts-*fabZ*), and *ΔfabZΔlpxH* at 30°C and 42°C.
