# Supplementary material for: Conditional lethality and suppressor analysis of plasmid-based temperature-sensitive fabZ expression in Pseudomonas aeruginosa
Source: J Biol Chem. 2025 Apr 26;301(6):108553. doi: 10.1016/j.jbc.2025.108553 (PMC12152623; doi:10.1016/j.jbc.2025.108553)
Supplement: Figure S3 [file mmc4.pdf]

A

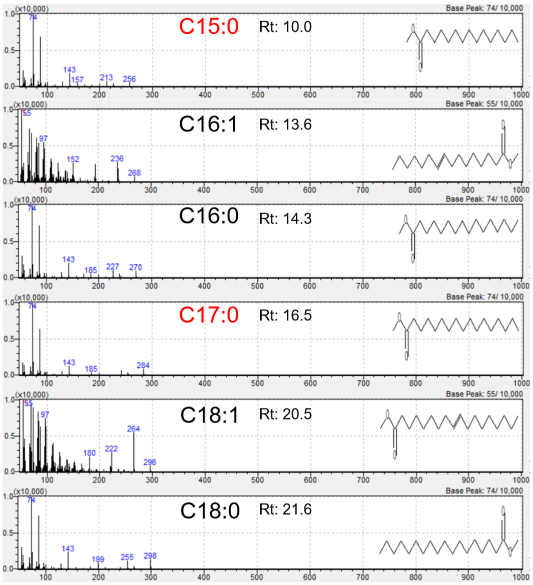

B

Relative content of fatty acid species based on GC-MS analysis of FAME

Strain	Fatty acid	Retention time	Peak Area	Relative Content
PAO1 (wt)	15:0	N/A	N/A	N/A
	16:1	13.6	8375142	7.5%
	16:0	14.3	53329965	47.7%
	17:0	N/A	N/A	N/A
	18:1	20.5	31588393	28.3%
	18:0	21.6	18473346	16.5%
ΔfabZΔlpxH	15:0	10.0	8921504	12.2%
	16:1	13.6	2251155	3.1%
	16:0	14.3	28201696	38.6%
	17:0	16.5	6903937	9.5%
	18:1	20.5	5780969	7.9%
	18:0	21.6	20975899	28.7%

Supplementary Figure S3. MS spectra and relative content of fatty acid species of FAME in *ΔfabZΔlpxH* and wild type. (A) MS spectra of fatty acid species of FAME. (B) Relative content of fatty acid species based on GC-MS analysis of FAME.
